# Single-cell tracking reveals tumor-reactive T cell plasticity during melanoma TIL therapy

**DOI:** 10.1016/j.xcrm.2026.103051

**Published:** 2026-09-15

**Authors:** Michael T. Sandholzer, Clara Serger, Alessia G. Liner, Sarp Uzun, David König, Helen Thut, Reto Ritschard, Jonas D. Fürst, Andreas Zingg, Natalia Rodrigues Mantuano, Benjamin Kasenda, Katharina Glatz, Elisabeth A. Kappos, Matthias Matter, Andreas Holbro, Frank Stenner, Jakob Passweg, Nina Khanna, Lukas Jeker, Mascha Binder, Alfred Zippelius, Heinz Läubli

**Affiliations:** 1 Department of Biomedicine, University of Basel and University Hospital Basel, Basel, Switzerland; 2 Division of Medical Oncology, University Hospital Basel, Basel, Switzerland; 3 Department of Pathology, University Hospital Basel, Basel, Switzerland; 4 Department of Plastic, Reconstructive, Aesthetic and Hand Surgery, University Hospital Basel and University of Basel, Basel, Switzerland; 5 Division of Hematology, University Hospital Basel, Basel, Switzerland; 6 Division of Infectious Diseases, University Hospital Basel, Basel, Switzerland; 7 Innovation Focus Cell Therapies, University Hospital Basel, Basel, Switzerland

**Keywords:** tumor-infiltrating lymphocytes, adoptive cell therapy, melanoma, cancer immunotherapy, T cell receptor repertoire, tumor-reactive T cells, T cell exhaustion, regulatory T cells, type 17 T cells, follicular helper T cells

## Abstract

Adoptive cell therapy with tumor-infiltrating lymphocytes (TILs) induces durable responses in metastatic melanoma, yet the clonal and transcriptional dynamics governing tumor-reactive T cell fate during *ex vivo* expansion and after transfer remain poorly understood. Here, we perform longitudinal single-cell RNA and T cell receptor sequencing across five time points, from baseline tumors through two-phase *ex vivo* expansion to post-infusion blood and tumor biopsies, in seven melanoma patients, resolving both the CD8^+^ and CD4^+^ compartments. Tumor-reactive CD8^+^ T cells are reinvigorated from exhaustion and acquire HLA-II-high or *KLF2*-high profiles. We further dissect the tumor-responsive CD4^+^ compartment in depth, revealing lineage-dependent reinvigoration in which follicular helper T cells adopt an effector state while exhausted CD4^+^ T cells retain dysfunction. In non-responders, across three cohorts, co-transferred type 17 T cells and *de novo* regulatory T cell expansion after transfer associate with treatment failure. These data define subtype-specific signatures across both lineages to guide TIL expansion.

## Highlights

• Single-cell RNA/TCR tracking maps tumor-reactive T cells across TIL therapy
• *Ex vivo* expansion reinvigorates exhausted tumor-reactive CD8^+^ T cells
• CD4^+^ Tfh adopt an HLA-II^+^ effector state while exhausted CD4^+^ cells persist
• Type 17 T cell co-transfer and Treg expansion link with TIL therapy failure

## Graphical abstract

**Figure:**
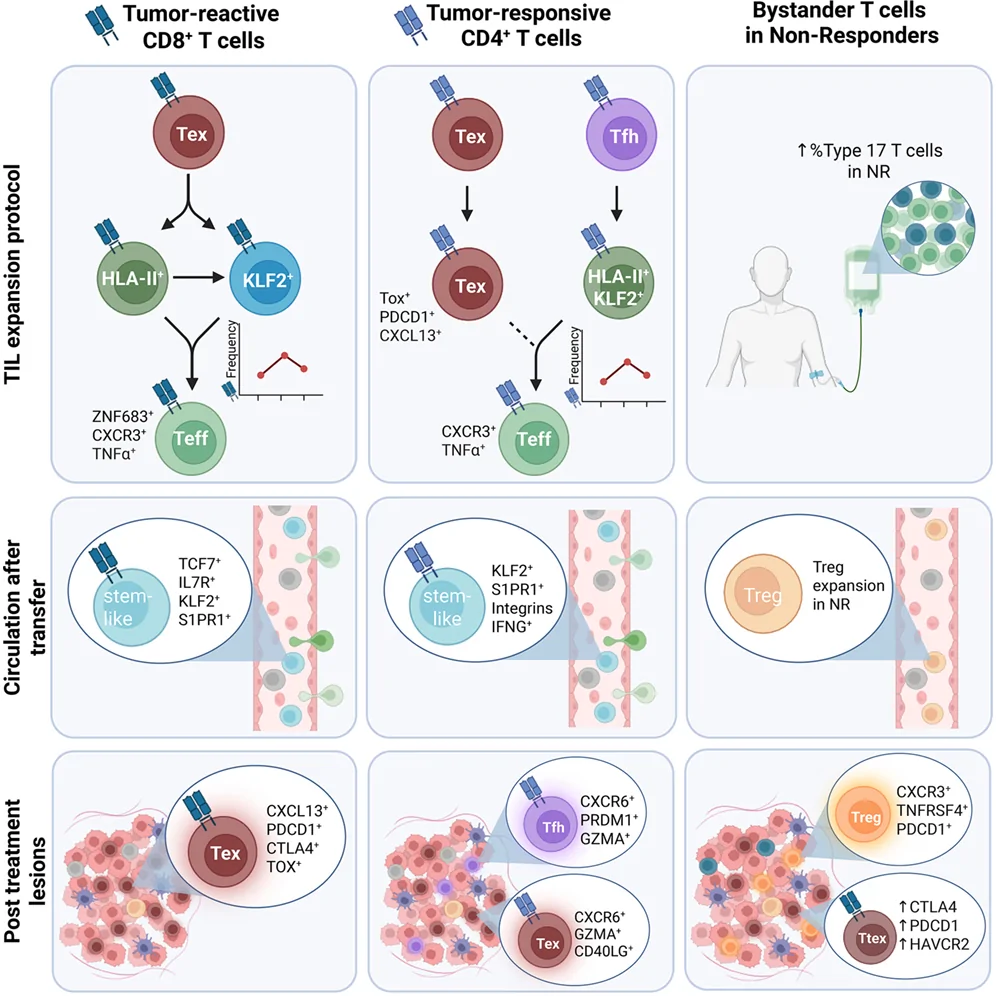

## Teaser

Sandholzer et al. track tumor-reactive T cells across TIL therapy in metastatic melanoma using single-cell RNA and TCR sequencing. They show how *ex vivo* expansion reshapes CD8^+^ and CD4^+^ T cell states and links type 17 T cell co-transfer and regulatory T cell expansion to treatment resistance.

## Introduction

Metastatic melanoma remains challenging to treat, given its aggressiveness, capacity for immune evasion, and marked intra- and intertumoral heterogeneity. Despite immune-checkpoint blockade (ICB) and BRAF/MEK inhibitors, durable responses are achieved only in a subset of patients.^1^^,^^2^ Adoptive cell therapy (ACT) using tumor-infiltrating lymphocytes (TILs) has emerged as a promising treatment for metastatic melanoma. Autologous T cells are isolated from tumor lesions, expanded *ex vivo*, and re-infused to enhance anti-tumor immunity, yielding objective response rates of up to 50% in heavily pre-treated patients.^3^^,^^4^^,^^5^ In a recent phase 3 trial, TIL-ACT more than doubled progression-free survival compared to ICB,^6^ leading to the FDA’s first approval of a commercial TIL therapy for patients refractory to standard therapies.^7^ Yet, only a subset of patients derives long-lasting benefit. A critical determinant of ACT efficacy is the ability of transferred T cells to persist *in vivo*.^8^^,^^9^ Studies have linked T cell persistence and anti-tumor efficacy to a less differentiated T cell phenotype with stem-like features, whereas fully differentiated effector T cells exhibit reduced persistence and impaired anti-tumor efficacy upon infusion.^10^^,^^11^^,^^12^ While ACT approaches using engineered T cell receptors (TCRs) or chimeric antigen receptors (CARs) address this by transferring less differentiated cells, TIL-ACT offers a distinct and complementary advantage by leveraging the natural polyclonality of tumor-infiltrating T cells, enabling broad tumor recognition, even in the face of tumor antigen heterogeneity.^13^^,^^14^^,^^15^ However, during the *ex vivo* expansion, tumor-dominant T cell clones are often outcompeted by newly emerging clones, reflecting complex and poorly understood T cell dynamics during TIL expansion.^16^ To achieve the full therapeutic potential of TIL-ACT, it is critical to elucidate the mechanisms governing clonal dynamics, T cell persistence, and effector function.

In this study, we address these critical gaps by dissecting the dynamics and persistence of tumor-reactive T cell subpopulations during TIL therapy. Leveraging longitudinal samples from patients with advanced metastatic melanoma enrolled in the BaseTIL trial, we tracked tumor-reactive T cell clones throughout TIL therapy, examining their fate during *ex vivo* expansion, post-transfer in the peripheral blood, and in post-treatment metastatic lesions.

## Results

### Plasticity and clonal dynamics of tumor-reactive CD8^+^ T cells

To investigate T cell fate decisions during TIL-ACT, we collected longitudinal samples from seven patients participating in the phase 1 BaseTIL trial (NCT04165967), which evaluated TIL therapy combined with the PD-1 inhibitor nivolumab in metastatic melanoma.^17^ Our cohort comprised two patients achieving partial response (response evaluation in solid tumors, RECIST v.1.1, 12-week assessment), classified as responders (Rs), and five non-responders (NRs; two with progressive disease, three with stable disease). Patient samples were collected at five time points throughout TIL-ACT for longitudinal analysis (Figure 1A). The baseline tumor served as the TIL source (pre-ACT) (1). *Ex vivo* expansion follows a two-step protocol^18^: tumor fragments are first cultured in high-dose IL-2 to generate an intermediate product (2) enriched for tumor-infiltrating T cells (pre-rapid expansion protocol, preREP); this product then undergoes a REP using high-dose IL-2, anti-CD3 antibodies, and allogeneic feeder cells, yielding the final TIL product (3). TILs are then infused into lymphodepleted patients with IL-2 support. After transfer, we collected peripheral blood mononuclear cells (PBMCs) 7 days post-transfer (7 dpt) (4), and from five patients metastatic lesions post-transfer (post-ACT) (5) (Table S1).

**Figure 1:**
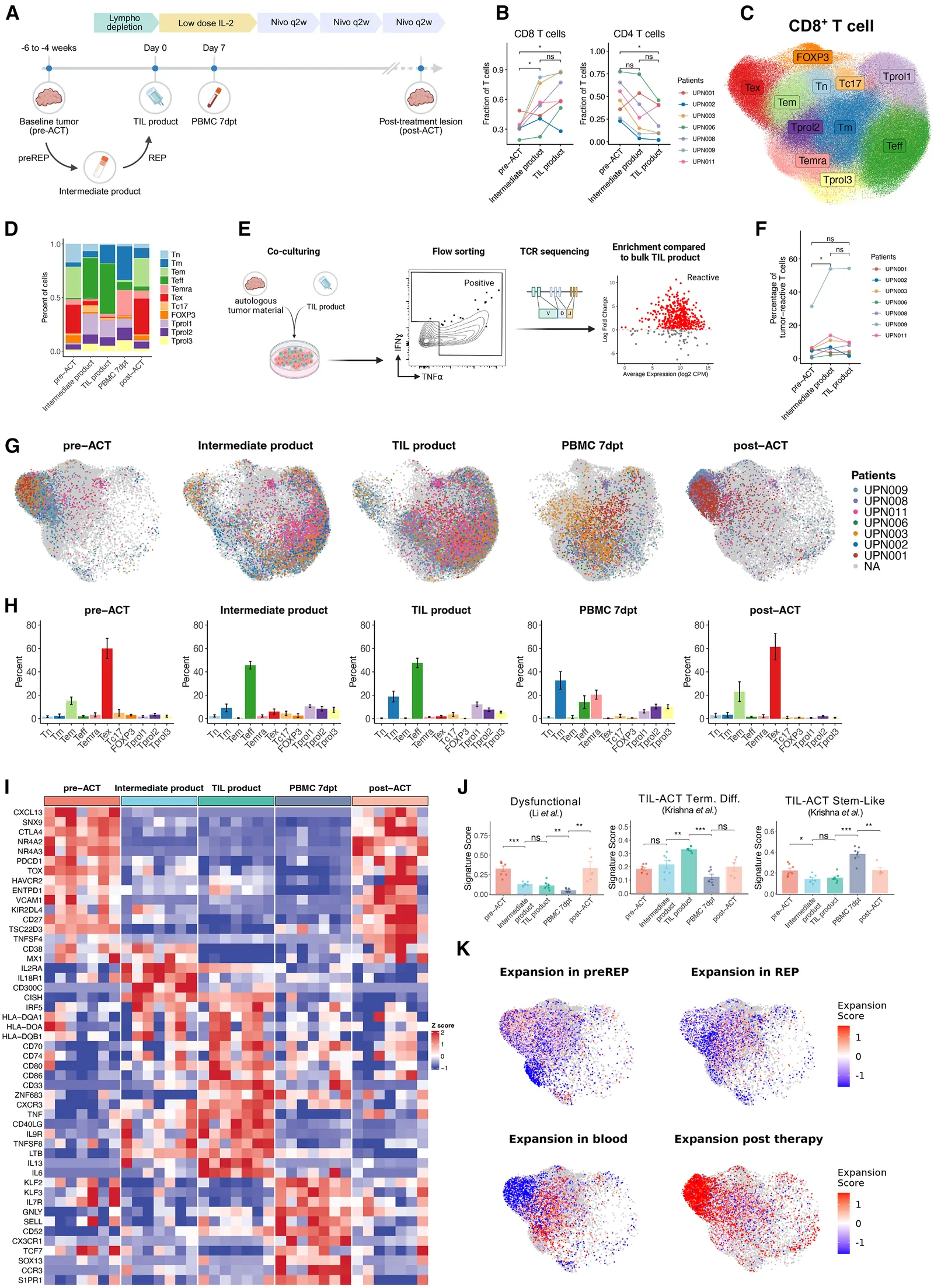
Plasticity and clonal dynamics of tumor-reactive CD8^+^ T cells (A) Treatment outline and sample collection time points: baseline tumor (pre-ACT); intermediate product; TIL product; peripheral blood mononuclear cells 7 days post-transfer (PBMC 7 dpt); post-treatment lesion (post-ACT). REP, rapid expansion protocol. Created with BioRender.com. (B) CD8^+^ and CD4^+^ T cell frequencies during TIL expansion; Wilcoxon signed-rank test. (C) UMAP visualization of CD8^+^ T cells. Clusters were annotated using their functional annotation or representative genes: Tn, naive T cells; Tm, memory T cells; Tem, effector memory T cells; Teff, activated effector T cells; Temra, terminally differentiated effector memory or effector T cells; Tex, exhausted T cells; Tc17, type 17 CD8^+^ T cells; FOXP3, CD8^+^ T cells expressing FOXP3; and Tprol, T cells with high expression of proliferation markers. (D) Proportional changes of CD8^+^ T cell subtypes during TIL therapy. (E) Graphical summary of tumor-reactive T cell identification. Created with BioRender.com. (F) Percent of tumor-reactive T cells during TIL expansion; Wilcoxon signed-rank test. (G) Tumor-reactive CD8^+^ T cells highlighted in the UMAP representation at collected time points. (H) Bar plots showing the mean CD8^+^ T cell composition of the tumor-reactive subpopulation per time point. Error bars indicate SEM. (I) Heatmap of the top differentially expressed genes in tumor-reactive CD8^+^ T cells between time points. (J) Signature scores of patient-bulked tumor-reactive CD8^+^ T cells during TIL expansion and post-transfer^12^^,^^19^; Wilcoxon rank-sum test. (K) Expansion score highlighted in the UMAP representation of pre-ACT or post-ACT. ∗*p* ≤ 0.05, ∗∗*p* ≤ 0.01, and ∗∗∗*p* ≤ 0.001. Unless indicated otherwise: pre-ACT *n* = 7; intermediate product *n* = 7; TIL product *n* = 7; PBMC 7 dpt *n* = 7; post-ACT *n* = 5 + 4. (B and F) *n* = 7 patients. (I) post-ACT *n* = 5 + 2. (J) post-ACT *n* = 5.

Isolated T cells from these samples were subjected to single-cell RNA-sequencing (scRNA-seq) and TCR sequencing (scTCR-seq). After filtering, high-quality expression data were obtained from 353′728 T cells, with TCRβ CDR3 regions in 284′339 cells (Tables S2 and S3). After dimensionality reduction, batch correction, and Leiden clustering, CD3^+^ T cells were separated into CD4^+^ and CD8^+^ subsets. During TIL expansion protocol, CD8^+^ T cells preferably expanded while CD4 T cells account for a reduced fraction in TIL products (Figure 1B). uniform manifold approximation and projection (UMAP) of CD8^+^ T cells revealed eleven subpopulations, annotated using published transcriptional signatures^20^ and marker genes (Figure 1C; Figures S1A and S1B).

Given the high clonal overlap and stable clonal hierarchy across time points, cluster analysis indicated substantial population shifts during TIL therapy: exhausted (Tex) and effector-memory (Tem) phenotypes in pre-ACT converged to effector (Teff) and proliferating (Tprol) profiles during expansion, while naive T cells (Tn) diminished, indicating activation and differentiation during REP (Figure 1D; Figures S1C and S1E). Seven days post-transfer, during IL-2 administration, Teff proportions were markedly reduced and most T cells displayed Tprol, Tm, or terminally differentiated effector memory (Temra) profiles, suggesting retained proliferation but reduced activation. Upon reencounter of the TME in tumor lesions collected post-ACT, the distribution of T cell profiles resembled that of pre-ACT baseline samples, with prominent Tex and Tem populations (Figure 1D).

As tumor-reactive clones are critical for tumor control, we next focused on identifying these TCRs and tracing their fate in more detail throughout TIL therapy. Thus, we developed a tumor reactivity assay in which TIL products were co-cultured with autologous tumor-derived single-cell suspensions or short-term cultured primary melanoma cell lines including HLA class I/II blocking control (Figure 1E; Figures S1F and S1G). Subsequently, tumor-reactive clones were identified by comparing TCRβ sequences from sorted IFNγ^+^ or TNFα^+^ T cells to those from bulk TIL products, defining enrichment based on log-fold change. Tumor reactivity was thus defined functionally (cytokine production) and by TCR sequence identity, independent of transcriptional phenotype, with the TCR serving as a stable molecular barcode to track clones across time points. The identified T cells were further validated by concordance with clonal expansion and published tumor-reactivity transcriptional signatures^21^^,^^22^^,^^23^^,^^24^ (Figures S1I–S1M). The proportion of tumor-reactive T cells in pre-ACT samples varied across patients but was detected consistently at all time points (Figure 1F; Figure S1H). Tumor-reactive clones generally increased during preREP, then increased more slowly or declined during REP (Figure 1F). Consistent with findings by Chiffelle et al.,^24^ tumor-reactive T cell clones predominantly displayed a Tex profile in pre-ACT samples and were reinvigorated during TIL expansion (Figures 1G and 1H). Already in preREP they transitioned to an activated Teff state, which became more pronounced after REP; in the final TIL product most displayed Teff characteristics with a smaller Tm subset. By 7 dpt, the majority had transitioned to a Tm or Temra profile. Notably, in post-ACT lesions, the majority of tumor-reactive T cells reacquired Tex features, closely resembling their pre-ACT counterparts (Figures 1G and 1H).

Differential gene expression (DGE) analysis comparing tumor-reactive clones between time points confirmed that in pre- and post-ACT samples they highly expressed exhaustion-associated markers (*PDCD1*, *CTLA4*, *TOX*, *KIR2DL4*) (Figure 1I). During preREP, this exhausted profile was downregulated, with upregulation of *IL2RA*, *HLA-DOA*, and *CD74*, genes linked to activation, antigen processing and presentation, as well as inhibitors of effector function and proliferation (*CISH* and *CD300C*)^25^^,^^26^ suggesting limited expansion potential (Figure 1I). In the final TIL product, tumor-reactive clones showed elevated expression of co-stimulatory ligands (*CD80* and *CD86*), activation markers (*TNF* and *CD40LG*), and genes involved in tumor homing and tissue-residency (*CXCR3* and *ZNF683*)^27^ (Figure 1I). By 7 dpt, the same clones showed increased stemness (*TCF7*, *SELL*, and *IL7R*) and immune cell trafficking (*KLF2*, *S1PR1*, and CX3CR1) markers, suggesting active trafficking through lymphoid tissue^28^^,^^29^ (Figure 1I). These shifts in transcriptional profiles were further confirmed using publicly available gene signatures,^12^^,^^19^ highlighting plasticity from dysfunctional to terminal differentiated profiles during TIL expansion but stem-like features after transfer (Figure 1J).

These data revealed considerable T cell adaptation to different environments including T cell activation and proliferation but also inhibition throughout different steps of TIL therapy. We therefore calculated expansion or contraction of clones across consecutive samples at single-cell level (Figure 1K). This analysis revealed that during preREP, especially tumor-reactive T cells in Tex cluster expanded significantly, indicating an IL-2-induced expansion of already activated T cells. During the REP phase, clone-independent stimulation by anti-CD3 antibodies caused robust expansion of Tn and Tem clones while tumor-reactive clones showed reduced proportional gain as bystander T cell expansion outpaced that of tumor-reactive clones. Consistent with this, TCR richness declined during preREP and increased during REP, the latter likely reflecting non-specific expansion of rare bystander clones above the detection threshold of our assays rather than *de novo* clonal generation (Figure S1D). By 7 dpt, Tem and Temra clones dominated peripheral blood, with fewer tumor-reactive clones than in the TIL product (Figure 1K). This aligns with the acquisition of stemness and trafficking markers in peripheral blood, consistent with these clones exiting the circulation and infiltrating tissues. We therefore compared proportional changes between PBMCs at 7dpt and post-ACT lesions (Figure 1K). Notably, especially tumor-reactive clones infiltrated post-ACT lesions and were found highly expanded in a Tex state.

Taken together, these data demonstrate that tumor-reactive CD8^+^ T cells undergo substantial clonal and transcriptional alterations during *ex vivo* expansion and after reinfusion into the patient. Already during preREP, *ex vivo* expansion shifted them from a canonical exhausted state toward an activated effector state, but they were subsequently diluted by disproportional bystander expansion during REP. After transfer, these clones showed increased trafficking potential and a stem-like profile in peripheral blood, implicating lymph nodes in maintaining and differentiating progenitor Tex into effector states.^29^ Even though tumor-reactive CD8^+^ T cells successfully infiltrated and expanded in post-ACT lesions, they reacquire exhaustion features, highlighting the immunosuppressive effects of the TME.

### Tumor-reactive CD8^+^ T cells acquire distinct expression profiles during reinvigoration

Because tumor-reactive T cells expanded and were functionally reinvigorated during preREP yet were diluted during REP by bystander expansion, we next examined their expression profiles in intermediate products to identify features that could improve TIL expansion.

First, we performed DGE comparing tumor-reactive CD8^+^ T cells with non-tumor reactive populations to resolve their distinct expression profile (Figure 2A). Tumor-reactive CD8^+^ T cells were mainly characterized by high expression of genes associated with antigen processing and presentation (*HLA-DQA1*, *HLA-DOA*, and *CD74*), as well as co-costimulatory ligands (*CD80*, *CD86*, and *CD70*). While retaining co-inhibitory receptors (*PDCD1* and *CTLA4*) and inhibitors of T cell proliferation (*CD300C*),^25^ they lacked stemness features (*TCF7*, *IL7R*, *LEF1*, and *CCR7*), which may explain their proliferative disadvantage relative to naive bystanders during REP. Subclustering intermediate products of six patients revealed seven distinct clusters (Figure 2B), with tumor-reactive clones primarily populating clusters 1 and 4 (Figure 2C; Figure S2A). These clusters showed high clonal overlap, indicating clone-independent development in which the same clone can adopt both states (Figure 2D). Since cluster 1 is characterized by high expression of genes known for HLA-II antigen presentation and low levels of exhaustion markers, it may represent cells early after reinvigoration (Figure 2E). In contrast, cluster 4 is characterized by *KLF2* expression, which is inversely regulated by TCR signaling,^30^ and genes often linked to immune cell trafficking and migration (*S1PR1*, *SELL*, *CX3CR1*, and *CXCR1*), suggesting a subset of tumor-reactive T cells not recently engaged in antigen recognition (Figure 2E; Figure S2E).

**Figure 2:**
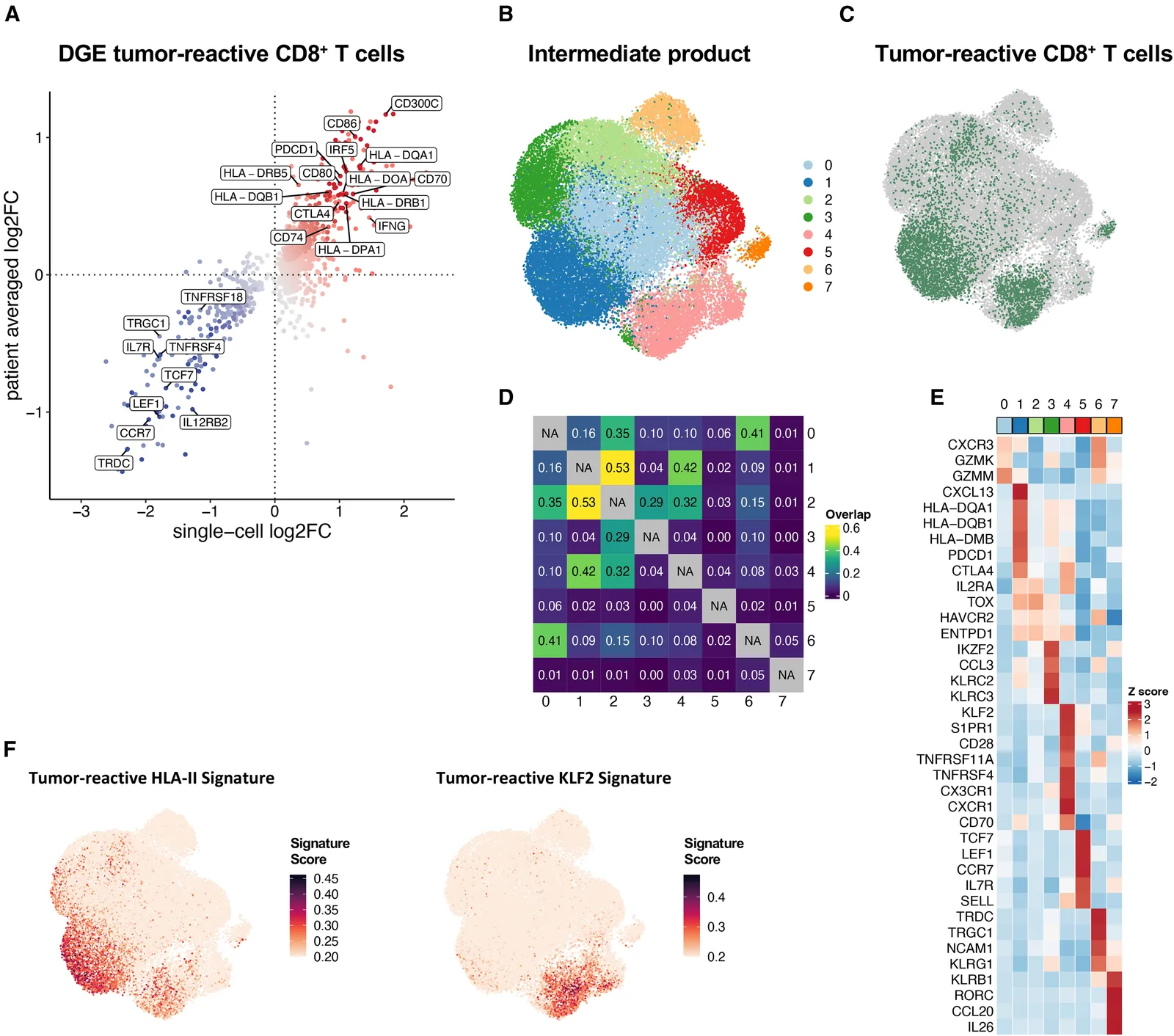
Tumor-reactive CD8^+^ T cells acquire distinct expression profiles during reinvigoration (A) Differential gene expression comparing tumor-reactive CD8^+^ subpopulations with bystander T cells in intermediate products using patient bulked comparison and single-cell comparison. Genes significantly upregulated in both comparisons were highlighted in dark color and genes significantly upregulated only in single-cell comparison in light color. (B) UMAP visualization of CD8^+^ T cell states in intermediate products. (C) Tumor-reactive CD8^+^ T cells highlighted in UMAP visualization of intermediate products. (D) Heatmap presenting clonal overlap between clusters. (E) Top expressed genes per cluster. (F) Tumor-reactive HLA-II and Tumor-reactive KLF2 Signature highlighted on UMAP. (A–F) *n* = 7 patients.

Finally, subtype-specific signatures confirmed that the two states represent distinct transcriptional profiles (Figure 2F; Figures S2B and S2C): clusters 1 and 4 both scored high for the tumor-reactive HLA-II signature, but only cluster 4 also scored high for the tumor-reactive KLF2 signature, suggesting a transition from an HLA-II high to a KLF2 high state.

In conclusion, tumor-reactive T cells with an exhausted profile in pre-ACT tumors downregulate exhaustion markers during culturing and IL-2 exposure in preREP, developing toward an HLA-II high state. Without a continuous TCR stimulus, for example once they grow out of tumor fragments or target cells die, they may transition to a KLF2 high state with features of increased immune trafficking. Both states can be defined by distinct signatures, allowing quantification or isolation during TIL expansion.

### Tumor-reactive CD8^+^ T cells exhibit dysfunctional profile in post-ACT of NRs

We next asked whether *ex vivo* expansion and transfer altered tumor-reactive T cell expression profiles after therapy, and whether reacquisition of exhaustion correlates with therapeutic outcome.

Therefore, we compared CD8^+^ tumor-reactive cells from pre-ACT lesions (*n* = 6) and post-ACT lesions (*n* = 5) from six patients, including two Rs and four NRs. Unsupervised clustering of 9,062 tumor-reactive CD8^+^ T cells revealed six distinct clusters (Figure 3A; Figures S3A–S3D). Trajectory inference suggested two predicted transcriptional continua originating from Tn and progenitor exhausted (Tpex) states: one toward ITGAE^+^ Tex and subsequently CXCL13^+^ Tex, the second toward GZMK^+^ Tex and then terminally exhausted T (Ttex) cells (Figure 3B; Figures S3E, S3F, and S3I). Comparing pre- and post-ACT lesions, NRs showed enrichment of terminally differentiated CXCL13^+^ Tex and Ttex with reduced Tn and Tpex, whereas Rs showed increased GZMK^+^ Tex, Tpex, and Tn (Figure 3C). Gene set enrichment analysis (GSEA) on matched clones confirmed this at single-clone level (Figures 3D–3F; Figures S3J and S3K). In NRs, transferred tumor-reactive clones showed increased expression of dysfunctional^19^ and terminally differentiated^31^ gene signatures, accompanied by a loss of stem-like features^31^ post-ACT (Figure 3D; Figures S3G, S3J, and S3K). In contrast, clones of a R retained stem-like T cell signatures with reduced dysfunctional^19^ and terminally differentiated^31^ profiles previously associated with favorable responses in immunotherapy^32^ (Figure 3F; Figures S3G, S3J, and S3K).

**Figure 3:**
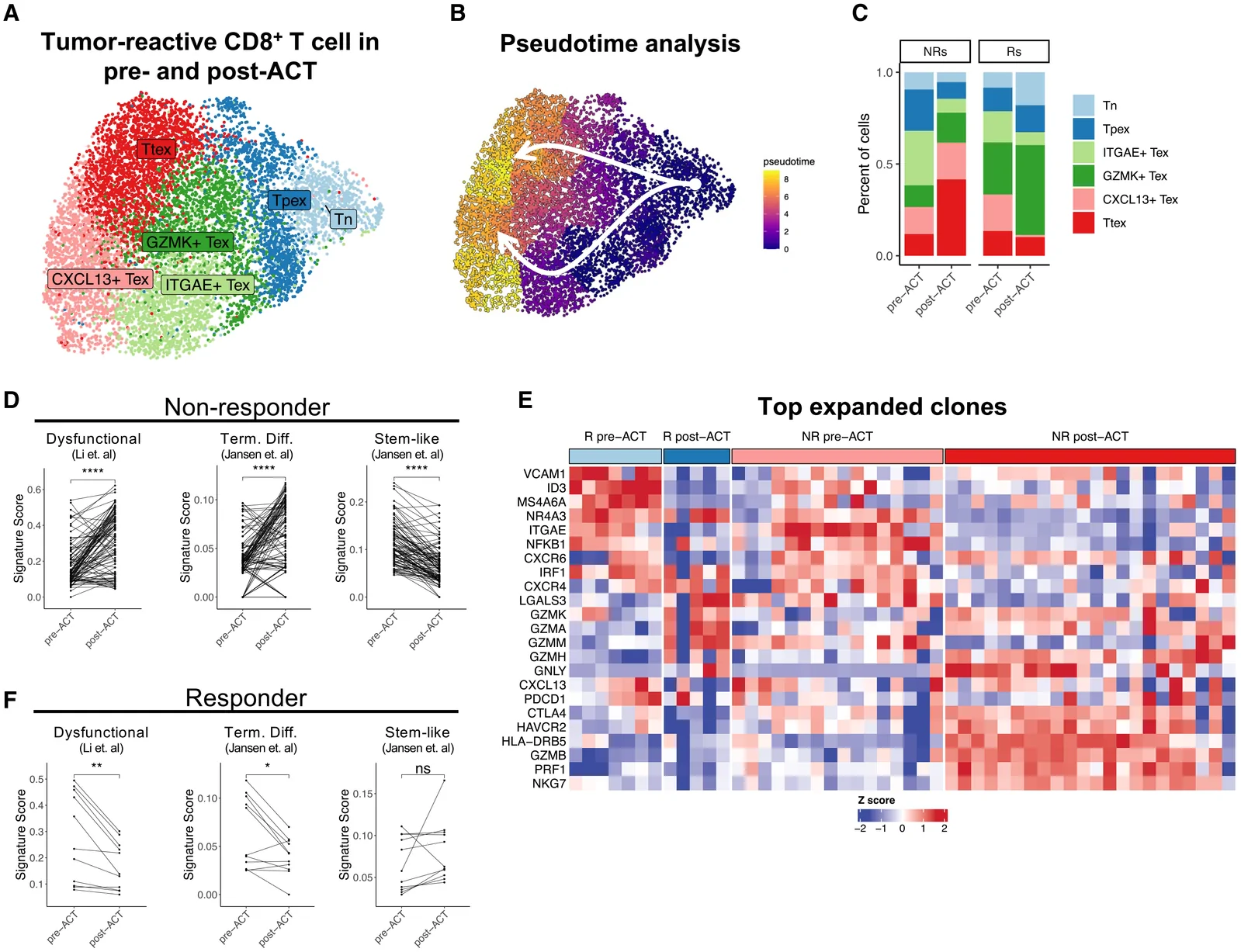
CD8^+^ tumor-reactive T cells show an elevated dysfunctional profile in post-ACT lesions of non-responders (A) UMAP visualization of tumor-reactive CD8^+^ T cell states in pre- and post-ACT lesions. Tpex, precursor exhausted T cells; Ttex, terminally exhausted T cells. (B) Pseudotime trajectories visualized on UMAP. (C) Cluster distribution of tumor-reactive CD8^+^ T cells detected in pre- and post-ACT lesions of responders (Rs) and non-responders (NRs). (D and F) Signature scores of published signatures comparing mapped clones of pre- and post-ACT lesions of Rs and NRs^19^^,^^31^; Wilcoxon signed-rank test. ∗*p* ≤ 0.05, ∗∗*p* ≤ 0.01, ∗∗∗*p* ≤ 0.001, and ∗∗∗∗*p* ≤ 0.0001. (E) Heatmap showing the mean relative expression of differentially expressed genes in expanded clones (>15 cells per clone) in pre- and post-treatment lesions of Rs and NRs. (A–C) pre-ACT *n* = 7; post-ACT *n* = 4 + 3. (D) *n* = 4 patients. (F) *n* = 1 patient. (E) *n* = 5 patients.

To further dissect transcriptional changes in tumor-reactive clones between pre-ACT and post-ACT samples of Rs and NRs, we performed DGE analysis on the top expanded clones (Figure 3E). Tumor-reactive clones in pre-ACT expressed markers of infiltration, tissue residency and early activation (*VCAM1*, *ITGAE*, *NR4A3*, *NFKB1*, and IRF1); post-ACT clones of the R mainly upregulated granzymes (*GZMK*, *GZMA*, *GZMM*, and GZMH), whereas NR clones showed high expression of inhibitory receptors and exhaustion markers (*PDCD1*, *CTLA4*, and HAVCR2), suggesting dysfunction (Figure 3E).

Overall, tumor-reactive CD8^+^ T cells in NRs are driven toward terminal exhaustion and dysfunction post-ACT, whereas Rs retain less differentiated T cells with cytotoxic effector signatures, underscoring the importance of maintaining early-differentiation states for effective TIL therapy.

### Plasticity and clonal dynamics of tumor-responsive CD4^+^ T cells

Having shown that tumor-reactive CD8^+^ T cells can undergo major transcriptional changes during TIL therapy, we next examined CD4^+^ T cells. Unsupervised clustering revealed nine distinct CD4^+^ T cell subsets (Figure 4A), which were annotated using signatures by Zheng et al.^20^ or identified by marker genes (Figures S4A and S4B). Similar to CD8^+^ T cells, CD4^+^ T cell subpopulations exhibited dynamic changes during TIL expansion and after transfer (Figure 4B). In pre-ACT lesions, CD4^+^ T cells primarily showed Tn, memory (Tm), T helper 17 (Th17), and regulatory (Treg) profiles (Figure 4B), transitioning during preREP and REP primarily to a Teff phenotype with increased Th17 and reduced Treg fractions. Seven days post-transfer, we observed a strong increase in Tregs and a decrease in Teff proportions in the peripheral blood. Notably, T cell profiles in post-ACT lesions largely mirrored those from pre-ACT samples but displayed higher fractions of Treg, Teff, and Th17 cells.

**Figure 4:**
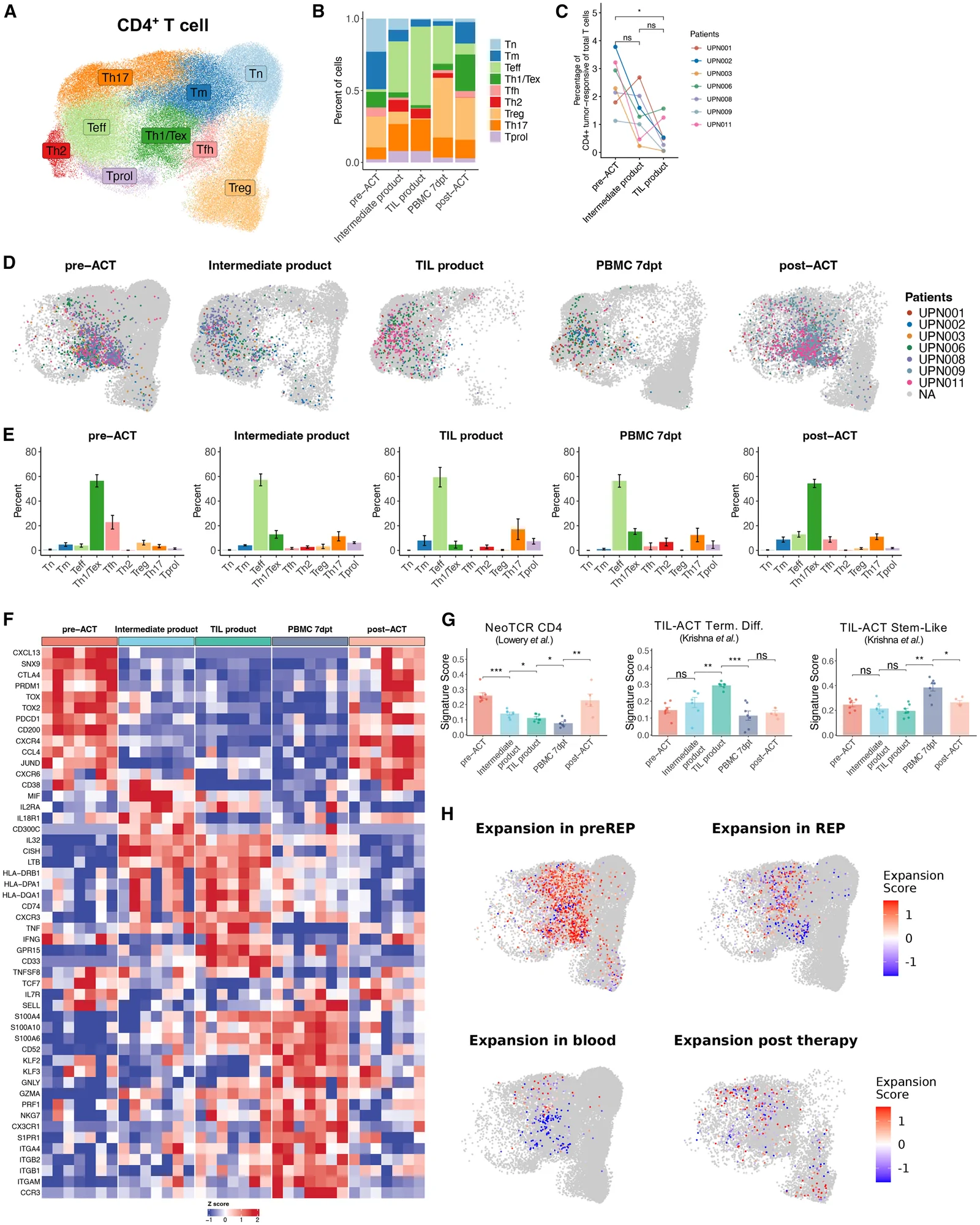
Plasticity and clonal dynamics of tumor-responsive CD4^+^ T cells (A) UMAP visualization of CD4^+^ T cell states. Clusters are highlighted using their functional annotation: Tm, memory T cells; Teff, activated effector T cells; Th1/Tex, T helper 1 cells and exhausted T cells; Tfh, T follicular helper cells; Th2, T helper 2 cells; Treg, regulatory T cells; Th17, T helper 17 cells; and Tprol, T cells with high expression of proliferation markers. (B) Proportional changes of CD4^+^ T cell subtypes during TIL therapy. (C) Percent of CD4^+^ tumor-responsive T cells of total T cells during TIL expansion; Wilcoxon signed-rank test. (D) Tumor-responsive CD4^+^ T cells highlighted in the UMAP representation at collected time points. (E) Bar plots showing the mean CD4^+^ T cell composition of the tumor-responsive subpopulation per time point. Error bars indicate SEM. (F) Heatmap of the top differentially expressed genes in tumor-responsive CD4^+^ T cells between time points. (G) Signature scores of patient-bulked tumor-responsive CD4^+^ T cells during TIL expansion and post-transfer^12^^,^^22^; Wilcoxon rank-sum test. (H) Expansion score highlighted in the UMAP representation of pre-ACT or post-ACT. ∗*p* ≤ 0.05, ∗∗*p* ≤ 0.01, and ∗∗∗*p* ≤ 0.001. Unless indicated otherwise: pre-ACT *n* = 7; intermediate product *n* = 7; TIL product *n* = 7; and PBMC 7 dpt *n* = 7; post-ACT *n* = 5 + 4. (C) *n* = 7 patients. (F) post-ACT *n* = 5 + 2. (G) post-ACT *n* = 5.

Although CD4^+^ T cells can be directly cytotoxic, most antitumor activity is expected to be indirect, via cytokine secretion, dendritic cell licensing, or support of CD8^+^ T cells.^33^ To capture the broader landscape of CD4^+^ T cells responding to tumor challenge (hereafter termed tumor responsive), we derived a transcriptional signature from tumor-responsive clones identified via our reactivity assay and applied it to conventional CD4^+^ T cells to identify additional clones with antitumor profiles resembling those directly tumor reactive. Using this signature, which associated with CD4^+^ T cell expansion and aligned with established neoantigen-reactive CD4^+^ T cell signatures^22^ (Figures S4C–S4F), we identified tumor-responsive clones and monitored their transcriptional dynamics during TIL therapy.

This approach revealed that tumor-responsive clones in pre-ACT mainly exhibited a Th1/Tex and follicular helper T cell (Tfh) profile (Figures 4D and 4E) and transitioned after preREP to a Teff profile, while only a fraction remained in a Th1/Tex state. During REP, nearly all tumor-responsive clones converged to a Teff state, while a small subset of Th17 expanded. However, during TIL expansion the total fraction of CD4^+^ tumor-responsive T cells reduced (Figure 4C). Seven days after TIL transfer, most tumor-responsive clones in blood retained an activated Teff state, while some reverted to a Th1/Tex profile (Figures 4D and 4E). Within post-ACT, the majority of tumor-responsive clones transitioned back to Th1/Tex, with only a fraction retaining the Teff state. Compared to pre-ACT, more clones acquired Teff, Th17, and Tm profiles and fewer a Tfh state, suggesting loss of tumor-responsive Tfh cells during TIL therapy (Figures 4D and 4E).

We next performed DGE comparing tumor-responsive CD4^+^ subsets between time points. As expected, tumor-responsive CD4^+^ clones expressed high levels of anti-tumor and exhaustion markers (*CXCL13*, *PDCD1*, and *TOX*) in pre-ACT, which were downregulated upon *ex vivo* expansion, suggesting reinvigoration. Similar to their CD8^+^ counterparts, in intermediate products, tumor-responsive CD4^+^ clones upregulated genes for HLA-II antigen presentation (*HLA-DRB1* and *CD74*) and markers for activated cytotoxic CD4^+^ T cells (*TNF*, *LTB*, and *IL2RA*), but also inhibitors of Teff function and proliferation (*CISH* and *CD300C*).^25^^,^^26^ Some transcripts were even more pronounced in TIL products where high expression of *TNF*, *IFNG*, and *CXCR3* suggests high activation and tumor homing potential (Figure 4F). Seven days post-transfer, the same clones expressed trafficking (*KLF2*, *S1PR1*, and *CX3CR1*), stemness (*TCF7*, *IL7R*, and *SELL*), and cytotoxicity (*GNLY*, *GZMK*, *PRF1*, and NKG7) markers, indicating a stemness reservoir with both tissue homing and effector potential (Figure 4F). In post-ACT lesions, tumor-responsive clones show again markers of anti-tumor response (*CCL4*, *TNF*, and *IFNG*), *CXCR6*, which is critical for sustained tumor control,^34^ but also immune checkpoints (*PDCD1* and *CTLA4*) (Figure 4F). GSEA of the tumor-responsive subpopulation using published signatures^12^^,^^22^ confirmed that anti-tumor-associated programs were largely absent during *ex vivo* expansion, where cells displayed terminal differentiation profiles, but re-emerged in peripheral blood with a stem-like expression signature (Figure 4G).

We next examined how distinct tumor-responsive CD4^+^ subsets expand or contract at the clonal level. Especially tumor-responsive Th1/Tex and Tfh subsets underwent significant expansion in the preREP phase but contracted in the REP phase (Figure 4H). During this, also Th17 cells expanded in preREP and partially in REP phase explaining increased fraction in final TIL products (Figure 4B). After transfer, similar to their CD8^+^ counterparts, also tumor-responsive CD4^+^ clones disappeared from circulation implying active tissue homing (Figure 4H). However, contrary to our expectations in post-ACT mainly Th17 or Treg subpopulations infiltrated from circulation and expanded, underscoring loss of beneficial tumor-responsive CD4^+^ T cells during TIL therapy.

Finally, we re-analyzed the dataset by Chiffelle et al.,^24^ using our signature to identify tumor-responsive CD4^+^ clones in pre-ACT and TCR sequences as barcodes to estimate their frequency in TIL products across that larger cohort. Although not statistically significant, Rs received on average a higher proportion of tumor-responsive CD4^+^ T cells than NRs, suggesting a potential contribution to the anti-tumor response (Figures S4K–S4N).

Together, *ex vivo* expansion markedly remodels tumor-responsive CD4^+^ clones, shifting them from an exhausted anti-tumoral profile toward cytotoxic and antigen-presenting profiles with enhanced tumor-homing potential. Similar to tumor-reactive CD8^+^ T cells, tumor-responsive CD4^+^ clones expanded efficiently during the first phase but became diluted in the second phase. Following adoptive transfer, these clones displayed stemness, tissue homing, and cytotoxic potential, but showed limited tumor infiltration. Notably, Th17 subpopulations expanded during *ex vivo* expansion and infiltrated along with Tregs post-treatment lesions, with potential implications for the anti-tumor response.

### Tumor-responsive Tfh and Tex retain transcriptional effector profiles post-ACT

To further explore TIL therapy-induced changes in tumor-responsive CD4^+^ T cells and their impact on the anti-tumor response, we subclustered them from pre-ACT (*n* = 6) and post-ACT (*n* = 5) lesions from six patients, including two Rs and four NRs (Figure 5A; Figures S5A–S5C). In post-ACT, NRs showed fewer tumor-responsive Tfh cells and more cells transitioning to a Ttex state (Figure 5B), whereas Rs displayed no major changes, suggesting that Tfh loss together with a Ttex increase may be linked to resistance. Subsequent pseudotime analysis confirmed Ttex and subsets of Tex as the terminal populations, which also displayed the highest clonal expansion (Figures 5C and 5D). In NRs, tumor-responsive CD4^+^ cells shifted overall toward terminal subpopulations, from predominantly naive or Tfh profiles in pre-ACT to Th1 and Ttex states in post-ACT (Figure 5E).

**Figure 5:**
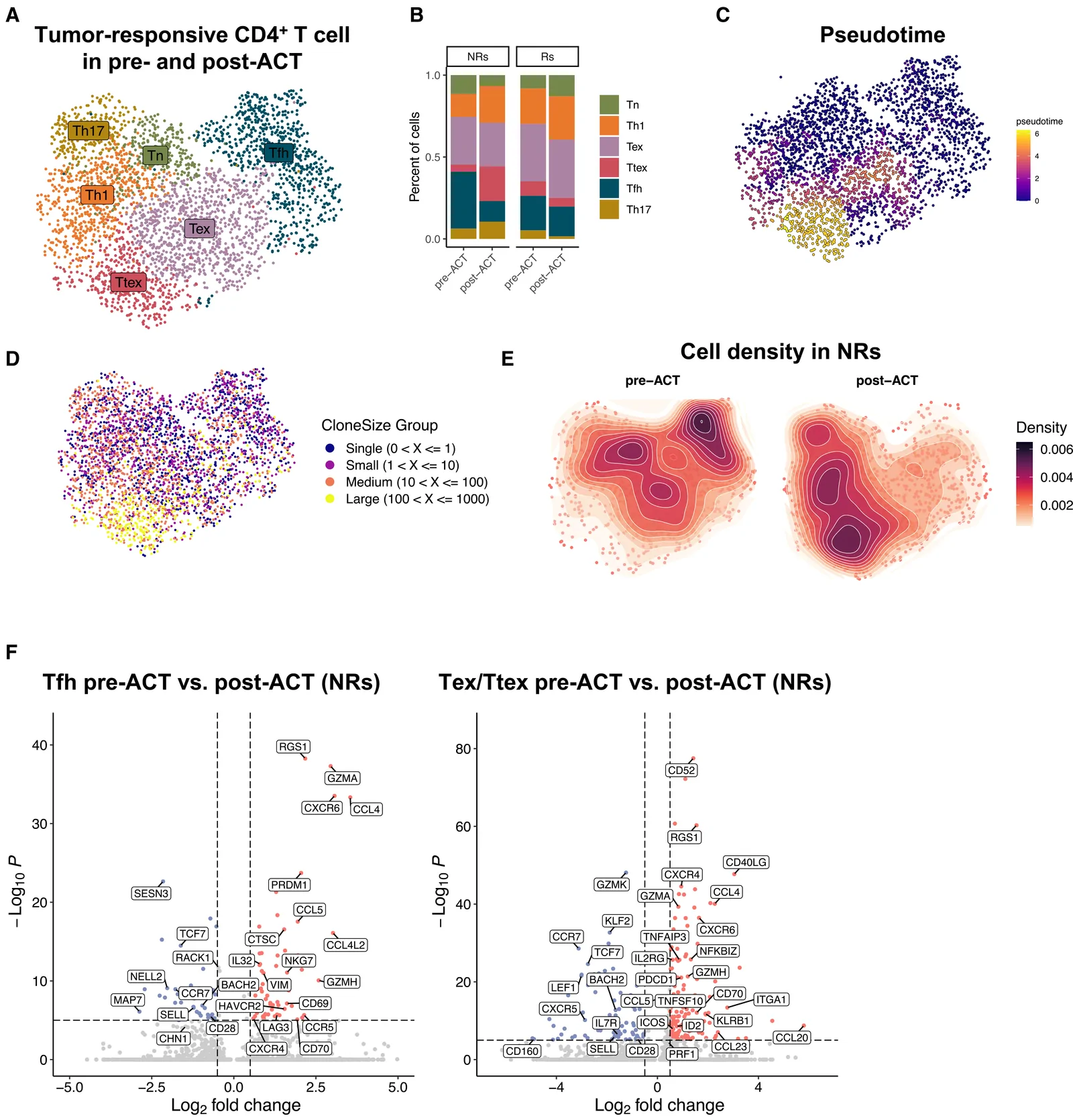
Tumor-responsive Tfh and Tex retain transcriptional effector profiles despite immunosuppressive TME (A) UMAP visualization of tumor-responsive CD4^+^ T cell states in pre- and post-ACT lesions. Th1, T helper 1 cells. (B) Cluster distribution of CD4^+^ tumor-responsive T cells detected in pre- and post-ACT lesions of responders (Rs) and non-responders (NRs). (C) Pseudotime scores visualized on UMAP. (D) Expansion state highlighted in the UMAP representation. (E) Cell density highlighted on UMAP of pre-ACT and post-ACT samples in NRs. (F) Volcano plot showing genes differentially expressed in tumor-responsive Tfh and Tex/Ttex comparing pre-vs. post-ACT in NRs. (A–E) pre-ACT *n* = 7; post-ACT *n* = 5 + 4. (F) *n* = 5 patients.

To examine transcriptional changes in the main shifting populations, we performed DGE on Tfh and Tex/Ttex comparing pre- and post-ACT in NRs. In both populations, DGE analysis revealed a loss of stemness markers (*TCF7*, *SELL*, and *CCR7*), an upregulation of Th1 markers (*CXCR6*, *PRDM1*, and *TNFSF10*) and cytotoxicity-associated genes (*CCL4*, *CCL5*, *GZMA*, and *GZMH*), indicating transition toward Th1-like effector states (Figure 5F).

Together, both tumor-reactive CD8^+^ and CD4^+^ T cells in NRs progressed toward terminal, more mature states: CD8^+^ T cells acquired a dysfunctional profile, whereas CD4^+^ T cells gained enhanced effector profiles with loss of stemness.

### Tumor-responsive Tfh but not Tex were efficiently reinvigorated during preREP

Having established that tumor-responsive CD4^+^ T cells retain effector potential in post-ACT lesions, we next assessed whether preREP contributes to their reinvigoration, by comparing them with other CD4^+^ T cells in intermediate products. Tumor-responsive clones not only expressed high levels of HLA-II antigen presentation genes (*HLA-DRB1* and *CD74*) and co-stimulatory ligands (*CD80* and *CD70*) but also showed exhaustion markers (*TOX* and *PDCD1*) and inhibitors of proliferation (*CD300C*),^26^ pointing to reinvigoration accompanied by partial dysfunction (Figure 6A).

**Figure 6:**
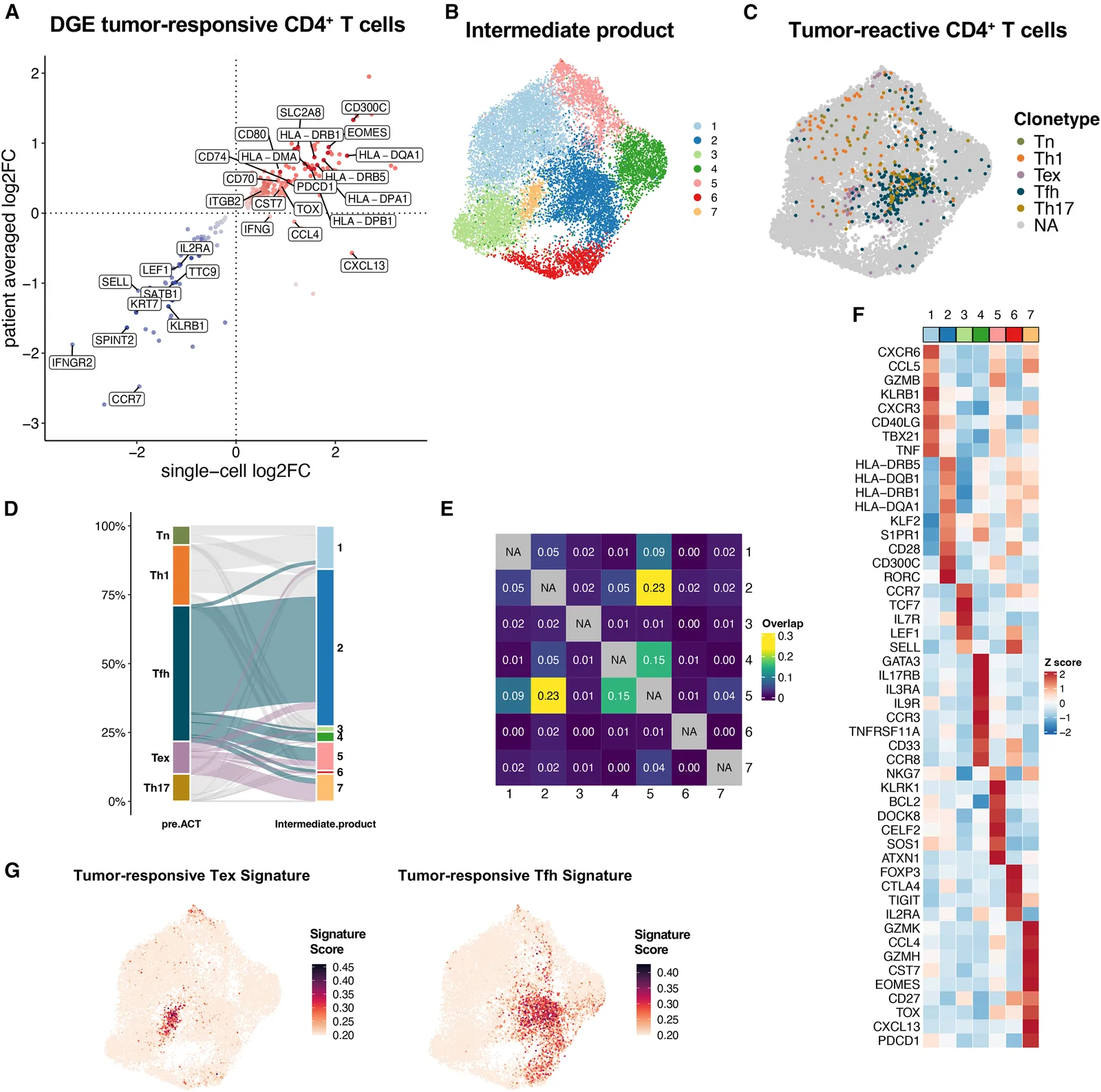
Tumor-responsive Tfh but not Tex were efficiently reinvigorated during preREP (A) Differential gene expression comparing tumor-responsive CD4^+^ subpopulations with bystander T cells in intermediate products using patient bulked comparison and single-cell comparison. Genes significantly upregulated in both comparisons were highlighted in dark color and genes significantly upregulated only in single-cell comparison in light color. (B) UMAP visualization of CD4^+^ T cell states in intermediate products. (C) Tumor-responsive CD4^+^ clone types highlighted in UMAP visualization of intermediate products. (D) Alluvial plot depicting cluster localization of tumor-responsive clone types identified in pre-ACT on intermediate product UMAP. (E) Heatmap presenting clonal overlap between clusters. (F) Top expressed genes per cluster. (G) Tumor-responsive Tex and Tumor-responsive Tfh Signature highlighted on UMAP. (A–G) *n* = 7 patients.

To resolve which profiles these clones adopt after preREP, we subclustered intermediate products from six patients, identifying seven CD4^+^ clusters (Figure 6B). Tfh-derived cells localized mainly in cluster 2, marked by enhanced HLA-II antigen presentation and migration (*KLF2* and *S1PR1*), resembling reinvigorated profiles similar to CD8^+^ T cells (Figures 6C–6F). In contrast, Tex-derived clones localized in cluster 7, characterized by cytotoxic activity (*CXCL13*, *GZMK*, and *CCL4*) but persistent exhaustion (*PDCD1* and *TOX*), suggesting inefficient reinvigoration (Figure 6F).

No clonal overlap was observed between clusters 2 and 7, indicating lineage-dependent rather than shared developmental trajectories during preREP (Figure 6E). DGE for Tfh and Tex subpopulations in intermediate products identified distinct signatures (Figures S6A and S6B) that, mapped onto the intermediate product UMAP, specifically highlighted reinvigorated Tfh and non-reinvigorated Tex populations without overlap (Figure 6G).

Together, high-dose IL-2 during preREP selectively reinvigorates Tfh but not Tex clones. The signatures identified here enable isolation and further characterization of these subpopulations.

### Co-transferred type 17 T cells are associated with resistance to TIL therapy

Having identified substantial *ex vivo* expansion and infiltration of Th17 in post-ACT lesions (Figures 4B and 4G), we next investigated their association with TIL therapy outcome.

Considering both CD4^+^ Th17 and CD8^+^ Tc17 cells, we observed an increased fraction of type 17 T cells in TIL products of NRs (Figure 7A). Subclustering of CD4^+^ and CD8^+^ T cells from TIL products of six patients similarly revealed enrichment of Th17 and Tc17 cells in NRs compared to Rs, suggesting a potential link between type 17 T cell abundance and resistance to TIL therapy (Figures 7B–7E; Figures S7A and S7B). Given the small number of Rs in our cohort, we reanalyzed TIL products from trials using comparable expansion protocols. In the cohort of Chiffelle et al.,^24^ TIL products from NRs likewise showed increased expression of type 17 T cell markers (*RORC*, *KLRB1, AQP3*, and *IL26)* and other Th17/Tc17-associated genes (Figure 7F; Figure S7E). Because subclustering did not resolve type 17 T cells there, we applied signatures derived from our cohort, corroborating the association of elevated type 17 T cells in TIL products with non-response (Figure 7G; Figure S7F). Similarly, reanalysis of bulk RNA-seq data from Thompson et al.^35^ showed that CD4^+^ TIL products from NRs were enriched in genes associated with activated type 17 T cells, supporting a link between type 17 T cells and non-response (Figure 7H; Figure S7I).

**Figure 7:**
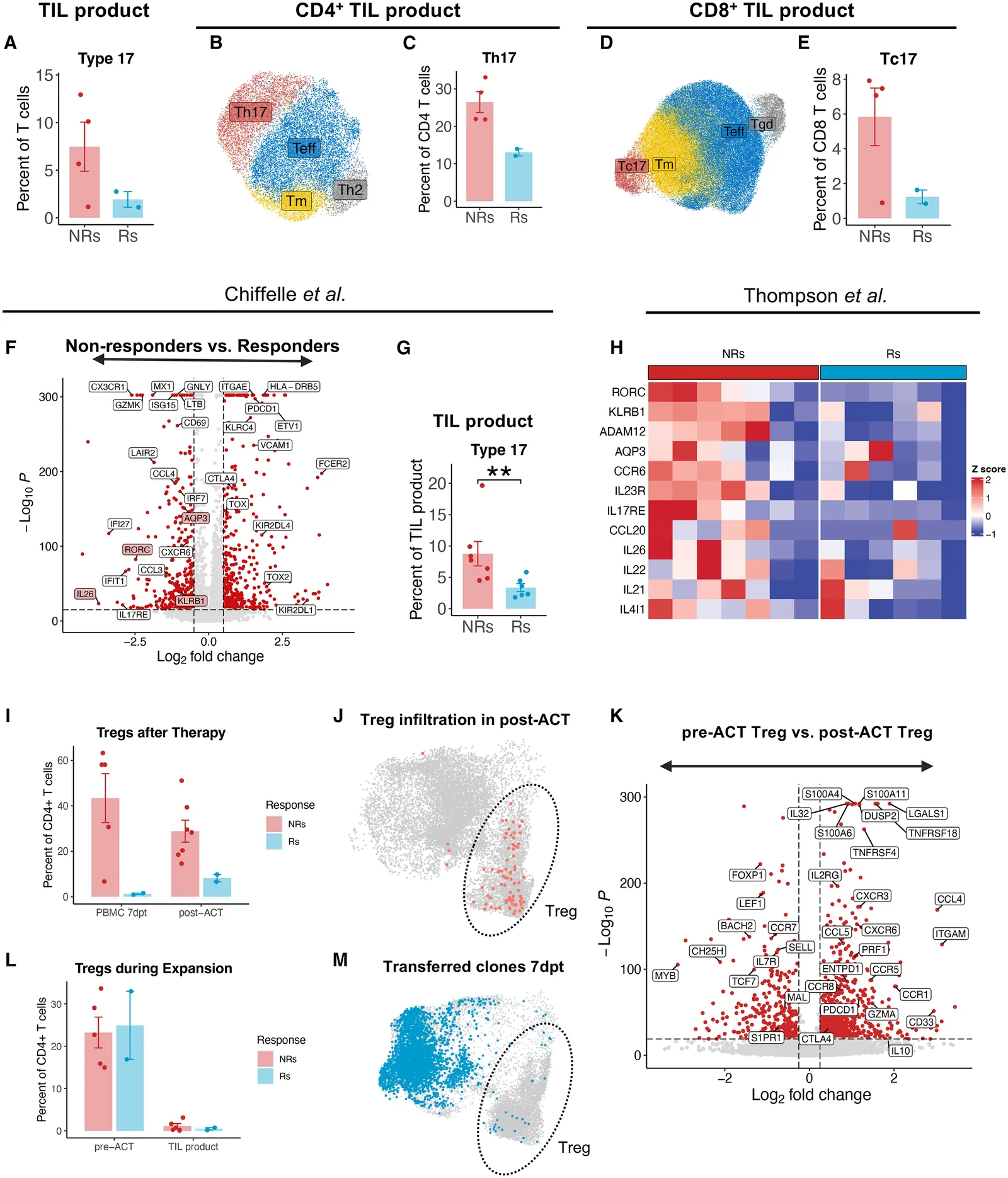
Co-transfer of type 17 T cells and Treg expansion post-transfer is associated with resistance to TIL therapy (A) Frequency of type 17 cells in TIL products comparing NRs and Rs. Error bars indicate SEM. (B) UMAP visualization of CD4^+^ TIL product. (C) Frequency of Th17 cells in CD4^+^ TIL products. (D) UMAP visualization of CD8^+^ TIL products. Tgd, gamma/delta T cells. (E) Frequency of Tc17 cells in CD8^+^ TIL products. (F) DGE comparing TIL products of NRs (*n* = 7) and Rs (*n* = 6) on dataset by Chiffelle et al.^24^ (G) Mean frequency of type 17 T cells in the TIL product in the dataset by Chiffelle et al.^24^ Error bars indicate SEM; Wilcoxon rank-sum test. ∗*p* ≤ 0.05, ∗∗*p* ≤ 0.01, and ∗∗∗*p* ≤ 0.001. (H) Heatmap showing the expression of selected marker genes expressed by Th17 and Tc17 in the dataset by Thompson et al.^35^ with seven NRs and six Rs. (I) Bar graph showing mean frequencies of Tregs in PBMC 7 dpt and post-ACT. (J) Clones of Tregs found in PBMC 7 dpt projected onto the UMAP of CD4^+^ T cells in post-ACT lesions. (K) Volcano plot showing genes differentially expressed in Tregs comparing pre-vs. post-ACT in NRs. (L) Bar graph showing mean frequencies of Tregs in pre-ACT and TIL products. Error bars indicate SEM throughout. (M) Clones of bulk TCR-seq of TIL drug product projected onto the UMAP in PBMC 7 dpt highlighting transferred clones. (A–E) *n* = 6 patients. (F–H) *n* = 13 patients.(I, J, L, and M) pre-ACT *n* = 7; post-ACT *n* = 5 + 4. (K) pre-ACT *n* = 5; post-ACT *n* = 4 + 3.

Overall, co-transferred type 17 T cells associate with resistance to TIL therapy in NRs across three independent datasets. Type 17 T cells were already present in baseline tumors, and TIL expansion protocols may inevitably promote their accumulation.

### NRs experience *de novo* expansion of peripheral Tregs after TIL transfer

Given the increased Treg accumulation in peripheral blood after TIL transfer despite their low numbers in TIL products, we next investigated their association with therapy outcome (Figure 4B). Notably, the accumulation of Tregs in PBMC 7 dpt was only observed in NRs (Figure 7I). Similarly, post-ACT lesions from NRs contained higher proportions of Tregs, whereas responding lesions showed reduced fractions (Figure 7I). Clonal tracking confirmed that peripheral Treg clones detected at 7dpt infiltrated post-ACT lesions in NRs (Figure 7J). To further characterize these newly infiltrated Tregs, we performed DGE analysis comparing pre- and post-ACT Tregs of NRs. Post-ACT Tregs in NRs upregulated chemokine receptors (*CXCR3*, *CXCR6*, *CCR5*, *CCR1*, and *CCR8*) and activation markers (*TNFRSF18*, *TNFRSF4*, *PDCD1*, and *ENTPD1*), indicating active recruitment, migration, and immunosuppressive activity within the TME (Figure 7K).

Further analysis suggested that pre-ACT lesions in NRs and Rs contained similar Treg proportions, and *ex vivo* expansion did not increase their numbers, indicating differential responses between Rs and NRs after TIL transfer (Figure 7L).

As Tregs were nearly undetectable in the final TIL products, we further investigated their origin to rule out differentiation of other subpopulations. TCR clonal overlap analysis between TIL products and PBMCs at 7 dpt showed that these Treg clones were largely undetected in the transferred product, even by bulk TCR-seq (∼500,000 T cells per sample), indicating *de novo* expansion from the endogenous repertoire rather than a TIL-product origin (Figures 7M and 7G).

These data suggest that early immunosuppression emerges post-transfer in NRs, with increased Treg accumulation in peripheral blood. Absent from the TIL products, these Tregs expanded *de novo* and exhibited tumor-homing features, potentially contributing to an unfavorable TME and diminished efficacy.

## Discussion

TIL-ACT has emerged as a promising approach for treating advanced melanoma, yet underlying T cell fate decisions during *ex vivo* expansion and resulting cellular composition of TIL products remain incompletely understood. Here, by leveraging longitudinal patient samples and complementary datasets, we characterize tumor-reactive subpopulations during TIL expansion and after administration, highlight potential reasons for therapy resistance, and offer actionable insights to improve TIL-based therapies.

Consistent with our findings, Chiffelle et al. reported that tumor-reactive CD8^+^ TILs are reinvigorated during TIL expansion, downregulating exhaustion markers.^24^ In post-ACT lesions these clones showed an exhausted effector state in Rs, whereas NRs showed poor infiltration with low cytotoxic activity.^24^ Our results confirm this plasticity and uncover additional transcriptional and clonal dynamics. At baseline, these clones predominantly exhibited an exhausted phenotype, consistent with other previous studies.^21^^,^^22^ During preREP, tumor-reactive clones preferentially expanded in response to IL-2, but their proportional representation decreased during REP, likely because polyclonal stimulation favors bystander expansion. Notably, during preREP expansion, exhausted tumor-reactive CD8^+^ T cells acquired distinct effector profiles characterized by high HLA-II expression, or, in the absence of recent TCR engagement, migratory profiles marked by high expression of *KLF2*. In contrast, the REP drove tumor-reactive CD8^+^ T cells toward a phenotype with increased cytotoxic and tumor homing potential. Post-transfer, tumor-reactive CD8^+^ clones in the peripheral blood exhibited stem-like profiles and expressed markers associated with lymph node egress consistent with the lymph node niche described by Tsui et al.,^29^ suggesting both tumor homing capacity and the potential to establish a stem-like reservoir. After infiltration into post-ACT lesions, however, these T cells reacquired expression patterns associated with both anti-tumor activity and exhaustion. Importantly, differences emerged between Rs and NRs: in Rs, tumor-reactive T cells retained earlier developmental states with stem-like features, whereas in NRs they shifted toward a terminally differentiated, dysfunctional phenotype with elevated expression of exhaustion markers. These findings differ from the observations by Chiffelle et al., but align with previous studies linking stem-like CD8^+^ T cells to response to ICB^32^ and underscore the importance of maintaining stem-like T cell states for optimal TIL-ACT efficacy. Of note, the trajectory analyses should be read as predicted transcriptional continua rather than formal differentiation pathways, as pseudotime inference can be unreliable without lineage tracing^36^; our predictions are nonetheless supported by TCR clonal and RNA velocity data.

While these findings highlight the dynamics of tumor-reactive CD8^+^ T cells during TIL therapy, tumor-responsive CD4^+^ T cells displayed distinct patterns that warranted separate investigation. In baseline tumors, two major subsets were observed: Th1 with their Tex counterpart and Tfh-like clones, the latter associated with tertiary lymphoid structure formation and favorable clinical outcomes across multiple solid tumors.^37^ Both subsets expanded during preREP; while Tex remained exhausted, Tfh were reinvigorated to an HLA-II-expressing state resembling that of tumor-reactive CD8^+^ T cells. During REP, tumor-responsive CD4^+^ clones, especially Tex and Tfh subpopulations with strong anti-tumor potential, were progressively outgrown by other T cells, although they acquired Teff characteristics including cytotoxic and tumor-homing markers. Post-transfer, these clones reappeared at lower frequencies in peripheral blood but in an activated stem-like state, expressing cytokines and migration markers suggesting active contribution to anti-tumor response. While tumor-reactive CD8^+^ clones were found exhausted and dysfunctional in post-ACT lesions of NRs, tumor-responsive CD4^+^ Tex shifted to more matured phenotypes with high cytotoxic potential. Notably, Tfh subpopulations also adopted a Th1-like phenotype characterized by elevated cytotoxic marker expression, suggesting TME remodeling potential despite ongoing exhaustion. Together, tumor-responsive CD4^+^ subsets may represent an underappreciated component of TIL therapy. We use the term tumor responsive rather than tumor reactive for the CD4^+^ compartment because activation may involve both direct recognition of MHC-II^+^ tumor cells and indirect presentation by intratumoral antigen-presenting cells, which are present in our heterogeneous autologous tumor suspensions. This is clinically relevant, as both directly reactive and helper CD4^+^ T cells contribute to the anti-tumor immune response *in vivo*.^33^

Additionally, we identified distinct signatures for tumor-reactive CD8^+^ and tumor-responsive CD4^+^ subpopulations within the intermediate product (after preREP). These signatures offer opportunities to prospectively identify and isolate relevant clones during *ex vivo* expansion. Enriching for tumor-reactive clones at this stage may reduce bystander T cell contamination improving overall TIL potency. Current phenotypic enrichment from tumors remains limited: markers, such as PD-1, CD39, and TIGIT associate with neoantigen reactivity, but these subsets often expand poorly *in vitro*,^38^ and early dysfunctional tumor-reactive T cells, despite greater proliferative potential, often lack these canonical markers.^39^ By contrast, during preREP we observed marked expansion of tumor-reactive subpopulations with distinct expression profiles, including *HLA-II*, *CD74*, *PDCD1*, *S1PR1*, *CD28*, and *CD70*. We propose leveraging these markers as enrichment markers in intermediate products, thereby enabling scalable isolation of tumor-reactive clones through approaches such as magnetic bead-based selection.^40^

Beyond tumor-reactive T cell dynamics, our study suggests that type 17 cells, not showing anti-tumor reactivity in our assays, were preferentially enriched in TIL products from NRs. This association was further supported by two independent publicly available datasets, in which type 17 T abundance also correlated with non-response. Although type 17 T cells can exert anti-tumor effects when their TCR recognizes tumor antigen,^41^ systemic IL-17 production can also promote tumor progression, inducing pro-survival and pro-angiogenic programs via IL-6-STAT3 signaling^42^ and recruiting immunosuppressive cells.^43^^,^^44^ Furthermore, Th17 cells directly promote Tregs activation, expansion, and stability via TNFR2 stimulation.^45^^,^^46^^,^^47^^,^^48^ Consistent with our findings, Knochelmann et al. showed that long-term expanded Th17 cells reduced therapeutic efficacy in mice by increasing Treg tumor infiltration.^49^ Together, these findings suggest an association between co-transferred type 17 T cells and reduced ACT efficacy, potentially through immunosuppressive circuits. These findings are correlative: elevated type 17 T cell levels may be an indirect biomarker of tumors low in tumor-reactive CD8^+^ T cells rather than a cause of treatment failure. Noting that treatment regimens differed across the analyzed trials, we propose assessing type 17 T cell abundance after *ex vivo* expansion as a candidate prognostic biomarker in larger cohorts.

Finally, our study underscores a potential role for Tregs in treatment resistance. IL-2 after TIL infusion is critical not only for engraftment and proliferation but also promotes Treg expansion, particularly at the low doses used in our trial.^50^^,^^51^ Peripheral Treg accumulation after TIL transfer has also been correlated with non-response under high-dose IL-2 regimens,^52^ suggesting a resistance mechanism shared across modalities. In our cohort, peripheral Treg expansion was observed specifically in NRs. These Tregs were largely undetected in the infused TIL product and appear to have expanded *de novo* from the patient’s endogenous repertoire rather than from the transferred TILs, exhibiting a strong immunosuppressive transcriptional profile. A link between transfer of high numbers of activated type 17 T cells in NR products and peripheral Treg accumulation at 7 dpt cannot be ruled out. Peripheral Tregs were enriched in post-ACT lesions of NRs, where they displayed a transcriptional profile associated with Treg activation, potentially contributing to impaired effector responses and heightened exhaustion of tumor-reactive T cells. Targeting this axis, for example with Treg-sparing IL-2 variants,^53^ could enhance TIL therapy efficacy.

Our findings identify several actionable strategies to improve TIL therapy efficacy. First, if Treg expansion is directly suppressive rather than a consequence of a failed CD8^+^ T cell response, limiting Treg expansion post-transfer may reduce progressive immunosuppression, particularly under low-dose IL-2. Treg-sparing IL-2 agents such as ANV419, currently in phase 1 evaluation (NCT05869539), offer a way to test this prospectively. Second, genetic editing targeting exhaustion regulators, including CISH, TOX, or NR4A can counteract transcriptional reversion to dysfunction upon tumor re-infiltration. Third, enrichment of tumor-reactive subpopulations during *ex vivo* expansion, leveraging the intermediate product signatures identified here, may enhance product potency and effector frequency at infusion. Fourth, prospective monitoring of type 17 T cell abundance after *ex vivo* expansion may provide a tractable biomarker of product quality and patient stratification, whether or not elevated type 17 levels are themselves suppressive. Incorporating these approaches into next-generation expansion protocols, alongside prospective studies with functional readouts, may enable more consistent and durable responses in metastatic melanoma.

### Limitations of the study

Several limitations should be considered. First, the relatively small, single-center cohort and inter-patient variability limit associations with clinical outcome; in particular, the links we highlight between co-transferred type 17 T cells, peripheral Treg expansion, and non-response are correlative and will require functional validation in larger cohorts. Second, the sampling depth of single-cell sequencing (typically 5,000–10,000 cells per sample) limits detection of rare populations, so we cannot formally exclude a contribution of stem-like progenitors below our detection threshold, although the high clonal overlap and stable clone hierarchy across time points argue against this as the sole explanation. Finally, post-transfer regimens differed across the cohorts compared here (e.g., IL-2 dosing and the addition of PD-1 blockade), which should be considered when generalizing across studies.

## Resource availability

### Lead contact

Further information and requests for resources and reagents should be directed to and will be fulfilled by the lead contact, Heinz Läubli (heinz.laeubli@unibas.ch).

### Materials availability

This study did not generate new, unique reagents. The autologous TIL products and patient-derived short-term cultured tumor cell lines (TCLs) generated in this study are patient-specific clinical materials and are not available for distribution.

### Data and code availability

• Single-cell RNA-sequencing and single-cell TCR-sequencing data have been deposited at the European Genome-phenome Archive (EGA) and at the Gene Expression Omnibus (GEO) and are publicly available as of the date of publication. Accession numbers are listed in the key resources table. Because these are human, patient-derived data, EGA access is controlled and is granted upon reasonable request following approval by the responsible Data Access Committee and completion of a data access agreement, consistent with the study’s ethics approval and the informed consent provided by participants.
• All original code has been deposited at GitHub and is publicly available as of the date of publication. The URL is listed in the key resources table.
• Any additional information required to reanalyze the data reported in this paper is available from the lead contact upon request.

## Acknowledgments

We thank the patients for allowing us to report their clinical information and data. We thank Werner Krenger and Anke Wixmerten for the support of the GMP productions. In addition, we thank Julia Manzetti, Dimitrios Tsakiris, and Astrid Tschan-Plessl for their initial help during the setup phase of the TIL program at the University Hospital Basel. We thank the Flow Cytometry Facility of the Department of Biomedicine at the University Hospital Basel, the Genomics Facility Basel and Christian Beisel for sequencing of the single-cell RNA libraries, and Petra Herzig for help with the patient samples. This work was supported by funding from the Fond’Action Foundation (to H.L.), by research support and drug supply of nivolumab by Bristol Myers Squibb, and by internal funding of the University Hospital Basel (Innovation Focus Cell Therapy). Research in the L.T. Jeker lab is supported by the 10.13039/501100000781European Research Council under the European Union’s Horizon 2020 Research and Innovation Program (grant agreement no. 818806).

## Author contributions

M.T.S., A.G.L., and S.U. performed the experiments; M.T.S. wrote the code and analyzed and visualized the data; H.T., R.R., A. Zingg., and N.R.M. performed GMP manufacturing of infusion products; H.L. and A. Zippelius provided supervision; M.T.S. and H.L. contributed to the experimental design; M.T.S., C.S., and H.L. prepared the manuscript; H.L. and A. Zippelius provided the funding; and H.L. and A. Zippelius led the research program.

## Declaration of interests

D.K. reports grants from Geistlich-Stucki-Stiftung; personal fees from Amgen, AstraZeneca, MSD, Novartis, Swiss Oncology in Motion, Mirati, Bristol Myers Squibb, Merck, and PharmaMar; personal fees and nonfinancial support from Amgen, Roche, and Sanofi, all outside the submitted work. A. Zingg reports grants from Bristol Myers Squibb during the conduct of the study. K.G. reports grants from Bristol Myers Squibb during the conduct of the study. L.J. reports grants, nonfinancial support, and other support from Cimeio Therapeutics outside the submitted work. F.S. reports personal fees from Bristol Myers Squibb and grants from Bristol Myers Squibb during the conduct of the study, as well as personal fees from Roche, Ipsen, AstraZeneca, MSD, and Takeda and grants from Takeda outside the submitted work. M.M. reports other support from Thermo Fisher Scientific, Merck, GlaxoSmithKline, Roche, Incyte, and Novartis outside the submitted work. A. Zippelius reports grants from Bristol Myers Squibb, and Fondaction during the conduct of the study, as well as grants from Roche, Bright Peak Therapeutics, T3 Pharma, and Astra-Zeneca outside the submitted work. H.L. reports grants from Fondaction and Bristol Myers Squibb during the conduct of the study, as well as grants and nonfinancial support from Bristol Myers Squibb; nonfinancial support from Merck Sharp Dohme; grants and personal fees from GlycoEra; and grants from Palleon Pharmaceuticals outside the submitted work. H.L. is a co-founder of Glycocalyx Therapeutics.

## Declaration of generative AI and AI-assisted technologies in the writing process

During the preparation of this work, the authors used artificial intelligence (AI)-assisted tools in order to improve the readability, language, and grammar of the text. After using these tools, the authors reviewed and edited the content as needed and take full responsibility for the content of the publication.

## STAR★Methods

### Key resources table

**Table:** 

REAGENT or RESOURCE	SOURCE	IDENTIFIER
**Antibodies**		

Anti-human CD3, BV421 (clone SK7)	BD Biosciences	Cat# 563797; RRID: AB_2744383
Anti-human CD45, PerCP-Cy5.5 (clone 2D1)	BD Biosciences	Cat# 340953; RRID: AB_400194
Anti-HLA-A,B,C (clone W6/32), purified	BioLegend	Cat# 311402; RRID: AB_314871
Anti-HLA-DR,DP, DQ (clone Tü39), purified	BioLegend	Cat# 361702; RRID: AB_2563139
Anti-human TNFα, APC (clone MAb11)	BioLegend	Cat# 502912; RRID: AB_315265
Anti-human IFNγ, BV421 (clone 4 S.B3)	BD Biosciences	Cat# 564791; RRID: AB_2738952
Human Fc Receptor Binding Inhibitor (eBioscience)	Thermo Fisher Scientific	Cat# 14–9161-73

**Biological samples**		

Melanoma metastasis resections (pre- and post-ACT)	University Hospital Basel (BaseTIL, NCT04165967)	N/A
Peripheral blood/PBMCs	University Hospital Basel (BaseTIL)	N/A
TIL infusion products and intermediate (preREP/REP) products	University Hospital Basel, GMP Facility	N/A

**Chemicals, peptides, and recombinant proteins**		

Accutase	PAA Laboratories	Cat# L11-007
Collagenase Type IV	Worthington Biochemical	Cat# LS004189
Hyaluronidase	Sigma-Aldrich	Cat# H3506
DNase type I	Sigma-Aldrich	Cat# DN25
Lymphoprep	STEMCELL Technologies	Cat# 07851
Propidium iodide	Thermo Fisher Scientific	Cat# P3566
CFSE (CellTrace)	Thermo Fisher Scientific	Cat# C34554
Recombinant human IFNγ (Imukin)	Boehringer Ingelheim	N/A
Monensin Solution (1,000×)	BioLegend	Cat# 420701
Brefeldin A Solution (1,000×)	BioLegend	Cat# 420601
Zombie NIR Fixable Viability Kit	BioLegend	Cat# 423105
Paraformaldehyde (4%)	Thermo Fisher Scientific	Cat# J19943.K2
Saponin	Sigma-Aldrich	Cat# 47036
RNasin Plus RNase Inhibitor	Promega	Cat# N2611
Solu-Cortef (hydrocortisone sodium succinate)	Pfizer	N/A
Sodium pyruvate (100 mM)	Sigma-Aldrich	Cat# S8636
Penicillin/streptomycin	Thermo Fisher Scientific (Gibco)	Cat# 15140122
Fetal bovine serum (FBS)	Sigma-Aldrich	Cat# F7524
Human AB serum	Blutspendezentrum Basel	N/A
RPMI-1640 (with GlutaMAX)	Thermo Fisher Scientific (Gibco)	Cat# 61870036

**Critical commercial assays**		

Chromium Next GEM Single Cell 5'/V(D)J Reagent Kits v2	10× Genomics	Cat# PN-1000263/PN-1000252/PN-1000286/PN-1000215
AllPrep DNA/RNA FFPE Kit (50)	Qiagen	Cat# 80234
Ion Torrent NGS Reverse Transcription Kit	Thermo Fisher Scientific	Cat# A45003
Oncomine TCR Beta-SR Assay, RNA	Thermo Fisher Scientific	Cat# A39359
Ion Universal Library Quantitation Kit	Thermo Fisher Scientific	Cat# A26217
Agilent BioanalyzerHigh Sensitivity DNA Kit	Agilent	Cat# 5067–4626/5067–4627
SPRIselect DNA Size Selection Reagent	Beckman Coulter	Cat# B23318

**Deposited data**		

scRNA-seq and scTCR-seq data (this paper)	European Genome-phenome Archive (EGA)	EGA: EGAP50000000107; GEO: GSM9814380
Reanalyzed human melanoma TIL dataset	Chiffelle et al., Immunity^24^	GEO: GSE229861
Reanalyzed human melanoma TIL dataset	Barras et al., Sci Immunol 2024	GEO: GSE222448
Pan-cancer T cell reference atlas/signatures	Zheng et al., Science 2021 (ref.^20^); https://doi.org/10.1126/science.abe6474	GEO: GSE156728
Human reference genome GRCh38 (2020-A); V(D)J GRCh38 ensembl-7.1.0	10× Genomics/Ensembl	N/A
Original analysis code (this paper)	Zenodo	https://doi.org/10.5281/zenodo.21930896

**Experimental models: Cell lines**		

Autologous short-term cultured tumor cell lines (TCLs), patient-derived	This paper (University Hospital Basel)	N/A

**Software and algorithms**		

CellRanger v7.1.0	10× Genomics	RRID:SCR_017344
R v4.3.0	R Foundation	RRID:SCR_001905
Seurat v5.1.0	Satija lab	RRID:SCR_007322
Harmony (Seurat implementation)	Korsunsky et al.	RRID:SCR_022206
UCell v2.6.2	Andreatta & Carmona; Bioconductor	RRID SCR_027109
Nebulosa v1.15.0	Alquicira-Hernandez & Powell; Bioconductor	https://github.com/powellgenomicslab/Nebulosa
scRepertoire v1.7.2	Borcherding et al.	RRID: SCR_025691
immunarch	ImmunoMind	RRID: SCR_023089
scirpy v0.13.1	Sturm et al.; scverse	https://github.com/scverse/scirpy
DESeq2 v1.42.1	Love et al.	RRID:SCR_015687
velocyto.py v0.17.17	La Manno et al.	RRID:SCR_018167
scVelo	Bergen et al.	RRID:SCR_018168
Monocle3 v1.3.4	Trapnell lab	RRID:SCR_018685
MiXCR v4.4.1	MiLaboratories	RRID:SCR_018725
ggsignif (geom_signif)	CRAN	RRID:SCR_023047

**Other**		

BD FACSMelody cell sorter	BD Biosciences	N/A
BD FACSAria III/SORP Aria III cell sorter	BD Biosciences	N/A
Illumina NovaSeq 6000	Illumina	N/A
Ion GeneStudio S5 Prime Sequencer; Ion 550 Chip	Thermo Fisher Scientific	N/A
2100 Bioanalyzer Instrument	Agilent	N/A

### Experimental model and study participant details

#### Patient population and trial design

The results of this clinical trial have been reported by König et al.^17^ Patients were eligible if they were 18 years or older and had histologically confirmed, unresectable stage III or IV cutaneous melanoma with disease progression after at least one anti-PD-1 based treatment line and an additional BRAF inhibitor in patients with BRAF V600 mutation, an accessible metastasis for tumor collection, measurable disease according to the Response Evaluation Criteria in Solid Tumors (RECIST), version 1.1 at the 12-week assessment, and Eastern Cooperative Oncology Group (ECOG) performance status score of 0 or 1 (on a 5-point scale, with higher scores indicating greater disability). The key exclusion criteria were non-cutaneous melanoma and active or untreated brain or leptomeningeal metastases.

In this academic phase I trial, patients underwent surgical excision of a metastasis for the generation of the TIL product. Nine patients were scheduled for TIL treatment. The TILs were manufactured at the GMP Facility for Advanced Therapies of the University Hospital of Basel. If the generation of the TIL product was successful, patients started lymphodepleting chemotherapy (LD) intravenously with cyclophosphamide at a dose of 60 mg/kg for days −7 and −6 and fludarabine daily at a dose of 25 mg/m^2^ (maximum of 50 mg) for days −5 to −1. Patients received TIL infusion on day 0, followed by daily subcutaneous administration of aldesleukin (interleukin-2, IL-2) at a dose of 125′000 IE/kg for 10 days, with a 2-day break after the 5^th^ dose. On day 14, patients were started on intravenous nivolumab at a dose of 240 mg and were thereafter treated with the same dose every 2 weeks. Treatment with nivolumab was continued until the occurrence of disease progression or for a maximum duration of 2 years. Patients received support with granulocyte colony-stimulating factor (G-CSF) after TIL infusion and further supportive treatment during TIL-ACT as needed. Prophylaxis for herpes simplex virus and pneumocystis was administered after TIL-ACT for 3 and 6 months, respectively.

The primary endpoint was the safety of the study intervention by assessing adverse events. Key secondary endpoints were the objective response rate (ORR), progression-free survival (PFS), and overall survival (OS). Tumor regression occurred in most patients (7/9) at the first imaging scan performed approximately one month after TIL-ACT. The best radiographic response according to RECIST v1.1 was partial response (PR) in 2 patients (ORR 22%) and stable disease (SD) in 3 patients. Four patients had progressive disease (PD) on the first imaging scan. All the patients experienced disease progression at one point. The median PFS after TIL transfer was 2.2 months. Five patients received subsequent systemic therapies, including the use of combined PD-1/CTLA-4 inhibitors in three patients. The median OS from TIL transfer was 7.2 months, with 5 and 3 patients alive 6 and 12 months after TIL transfer, respectively. For translational purposes, we classified two patients with PR as responders (Rs), and two patients with PD and three patients with SD as non-responders (NRs).

All participants provided written informed consent prior to enrollment. The protocol and amendments were reviewed and approved by the local ethics committee (Ethikkommission Nordwest-und Zentralschweiz, EKNZ, Basel, Switzerland; EKNZ No. 2019–01908) and the Swiss Agency for Therapeutic Products (Swissmedic, Bern, Switzerland). The trial (ClinicalTrials.gov: NCT04165967) was conducted in accordance with the International Council for Harmonization Good Clinical Practice guidelines and the principles of the Declaration of Helsinki.

The patients analyzed here represent a subset of the BaseTIL trial cohort. Across the nine TIL-treated trial participants, the median age at TIL transfer was 61 years (range, 35–66) and the cohort comprised four female and five male patients (Koenig et al.^17^); per-patient sex and clinical response for the present single-cell cohort are reported in Table S1. Sex was recorded as the biological variable; gender identity, ancestry, race, ethnicity, and socioeconomic status were not collected as part of this trial and are therefore not reported.

### Method details

#### Patient material processing

Resected solid tumor lesions were mechanically dissociated, digested with accutase (PAA Laboratories), collagenase IV (Worthington), hyaluronidase (MilliporeSigma), and DNAse type I (MilliporeSigma), filtered, washed, and frozen as single-cell suspensions for future use. Human PBMCs were isolated from whole blood by density gradient centrifugation using LymphoprepTM (Stemcell). PBMCs were cryopreserved for subsequent use. Aliquots of the TIL infusion products were frozen directly after harvest and stored in liquid nitrogen for later use. Excess intermediate products were obtained via the TIL expansion process.

#### Fluorescence-activated cell sorting for single-cell RNA-sequencing

Cells were thawed, washed, and resuspended in staining buffer (PBS with 1% BSA) containing a Hu Fc Receptor Binding Inhibitor (Thermo Fisher Scientific). After 10 min of incubation at 4°C, surface stain solution containing antibodies (CD3 BV421 Clone: SK7; CD45 PerCP-Cy5.5 Clone: 2D1) was added and incubated for 30 min at 4°C. The cells were washed twice and resuspended in staining buffer containing propidium iodide (1:1000; Thermo Fisher Scientific). Fluorescence-activated cell sorting (FACS) was performed using BD Melody or BD FACS Aria III. The cells were gated on single cells, live (PI-negative), CD45^+^, and CD3^+^. Cells were sorted in cold PBS with 2% FBS (Sigma-Aldrich) and used immediately for the single-cell immune profiling protocol (10× Genomics).

#### Single-cell RNA and single-cell TCR sequencing

Single-cell TCR-seq and 5′ gene expression profiling of single-cell suspensions using the Chromium Single Cell V(D)J Reagent Kits v2 from 10× Genomics was performed according to the manufacturer’s instructions. Cells were loaded in a 10× Genomics cartridge to achieve a target recovery of approximately 10,000 cells for each sample. Cell-barcoded 5′ gene expression libraries and TCR libraries were sequenced using the Illumina NovaSeq6000 system by the Genomics Facility Basel. Gene expression and V(D)J reads were aligned in parallel and annotated using CellRanger v. 7.1. For this the human reference genome GRCh38 2020-A and human V(D)J reference genome GRCh38 ensembl-7.1.0. was used.

#### Quality control and normalization of scRNA-seq data

The read count matrices were processed and analyzed using R v4.3.0, and Seurat v5.1.0, with default parameters for all functions unless otherwise specified.^54^ Cells were filtered using nFeatures over 650, nCounts over 1250, mitochondrial gene percentage of less than 15%, ribosomal gene percentage of more than 5%, and hemoglobin gene percentage of less than 10%. Doublets were identified based on T cells containing more than one TCR-β chain and removed. Counts were normalized and regressed for cell cycle, percentage of mitochondrial genes, and interferon response genes.^55^

#### Dimensional reduction and clustering

Genes irrelevant for clustering, for example, TCR genes, histone genes and ribosomal genes were excluded from variable features by using the gene blacklist used by Zheng et al.^20^ After PCA reduction, Harmony integration implemented in Seurat was used to integrate the dataset and remove patient- and sample-type-specific alterations. The 20 first Harmony corrected PCs were used to calculate the UMAP representation. For clustering the 20 first corrected PCs were used as input for the Leiden algorithm implemented in Seurat with resolutions from 0.3 to 0.6. T cell subsets were separated based on *CD8A, CD8B*, or *CD4* expression per cluster. The clustering procedure was repeated as described above until no CD8 or CD4 specific clusters were detected. The datasets were further purified from cells expressing *CD8A*, *CD8B*, or *CD4*. Cell type-specific clusters were identified using canonical marker genes and signatures, as published by Zheng et al.^20^ The R package UCell v2.6.2 was used for signature scoring. The R package Nebulosa v1.15.0 was used for gene expression visualization on UMAPs.

#### scTCR-seq analysis

TCR count metrics were integrated into the Seurat object using scRepertoire v1.7.2. For quality control and data exploration purposes, the R packages scRepertoire v1.7.2, immunarch,^56^ and scirpy v0.13.1 were used. For clone tracking, clonotypes were defined based on the nucleotide sequence of the CDR3-β region. The expansion score was defined as the log2-fold change in the frequency at which a clone was detected in two subsequent samples. Specifically, for each clone, the expansion score was calculated as log2(frequency in sample B/frequency in sample A), where frequency is the proportion of a given clone among all cells with a detected TCR in that sample. An expansion score above zero indicates proportional expansion, while a score below zero indicates proportional contraction relative to other clones.

#### Differential gene expression

DGE was performed using the in Seurat implemented function FindMarkers with a log fold change threshold set to zero. For patient-averaged differential gene expression of tumor-reactive vs. bystander T cells, an aggregated count matrix was created, and the standard DESeq2 v1.42.1 workflow was followed using sample origin as a covariate.

#### RNA velocity trajectory analysis

Spliced and unspliced reads were counted using the velocyto.py package v0.17.17 from aligned bam files generated by CellRanger v7.1.0. TCR, histone, and ribosomal genes were excluded using the gene blacklist by Zheng et al.^20^ From the resulting count matrices top 2000 variable genes with a minimum of 20 shared counts were selected and normalized. The moments were calculated for velocity estimation and used in the dynamic model of scVelo to learn the unspliced/spliced phase trajectory. For visualization, the resulting trajectory vectors were embedded into the UMAP space.

#### Pseudotime trajectory analysis

For pseudo temporal analysis we used the R package Monocle 3 v1.3.4. First, we loaded the pre-processed Seurat object into Monocle. After size factor estimation and combining all cells into one partition, we used the learn_graph function with default parameters to learn the cell connectivity. Next, we ordered the cells using the naive T cell cluster Tn as root cells. We used spatial autocorrelation analysis implemented in Monocle 3 to infer differentially expressed genes along the pseudotime axis.

#### Expansion of TCL

Autologous short-term cultured tumor cell lines (TCLs) were generated separately from TILs, either from tumor digests, tumor fragments, or after mincing, as previously described.^57^^,^^58^^,^^59^ Adherent cells were passaged in RPMI 1640 medium supplemented with 10% FBS, 1 mM sodium pyruvate (Sigma-Aldrich), 100 U/mL penicillin, 100 μg/mL streptomycin (Thermo Fisher), and 500 ng/mL Solu-Cortef (Pfizer AG). To establish TCLs, freshly resected tumor material was enzymatically digested or mechanically minced into small fragments and plated in the medium described above in tissue-culture-treated flasks. Cultures were maintained at 37°C and 5% CO_2_, with medium changes every 3–4 days to remove non-adherent lymphoid and dead cells, and adherent tumor cells were allowed to grow out over approximately 2–6 weeks. Upon reaching about 80% confluence, adherent cells were detached with trypsin-EDTA and passaged; established lines were expanded to a maximum of 10 passages and cryopreserved.

#### Anti-tumor reactivity assay

To assess tumor reactivity, the co-culture assay described by Andersen et al.^58^ was adapted. Briefly, TILs were thawed and rested for two days in assay medium (RPMI-1640 plus GlutaMAX with 1 mM sodium pyruvate (Sigma-Aldrich), 100 U/mL penicillin, 100 μg/mL streptomycin (Thermo Fisher), and 10% heat-inactivated AB Human serum). The TILs were washed, CFSE-labeled (Thermo Fisher Scientific), and co-cultured at 37°C for 10 h with fresh tumor digest or autologous short-term cultured tumor cell lines (TCLs) at an effector/target (E/T) ratio of 1:1. The TCLs were pre-treated for 72 h with 100 IU/mL interferon-gamma (IFNγ; Imukin, Boehringer-Ingelheim). Monensin (BioLegend) and Brefeldin A (BioLegend) were added (dilution 1:1000 each) at the beginning of the co-culture. As negative controls, TCR-HLA interactions were blocked with anti-HLA-A, B, C (clone: W6/32, BioLegend, 20 μg/mL) and anti-HLA-DR, DP, DQ (clone: Tü39, BioLegend, 20 μg/mL).

#### Fluorescence-activated cell sorting for anti-tumor reactivity assay

For intracellular staining procedures while preserving high RNA quality, the instructions of Healy et al.^60^ were followed. First, cells were washed with PBS and stained with Zombie NIR (BioLegend) for live/dead labeling. After washing in PBS with 1% FBS, the cells were fixed and permeabilized in a solution containing 4% PFA (Thermo Fisher Scientific), 0.1% saponin (Sigma-Aldrich), and 1 unit/μL of RNasin Plus RNase inhibitor (Promega) on ice for 20 min in the dark. Cells were then washed with permeabilization buffer (0.1% saponin, 2 M NaCl (Thermo Fisher Scientific), 0.5% BSA (Sigma-Aldrich) in RNase-free PBS (Thermo Fisher Scientific)), and intracellular staining was performed in permeabilization-stain buffer (PBS with 0.1% saponin containing 1 unit/μL of RNasin Plus RNase inhibitor) supplemented with anti-TNF ⍺-APC (clone: MAB11, Biolegend) and anti-IFNɣ-BV421 (clone: 4 S.B3, BD Biosciences) at 4°C for 45 min. Intracellular staining was performed at a density of 10 × 10^6^ cells/mL. The stained cells were then washed twice with permeabilization buffer and resuspended in sorting buffer (1% BSA, 2 M NaCl in RNase-free PBS). TNF ⍺ or IFNɣ positive live populations were sorted using BD SORPAria III.

#### Total RNA isolation from sorted T cells

Cells were isolated from the sort buffer by centrifugation at 2000 RCF for 15 min at 4°C. Total RNA and DNA were extracted simultaneously using the AllPrep DNA/RNA FFPE Kit (Qiagen), with some modifications from the vendor’s instructions. Briefly, the deparaffinization step was skipped and directly followed by protein digestion, which was extended for 1 h at 56°C at 300 rpm. After the separation of total DNA and RNA, incubation at 80°C for partial reversion of formaldehyde modifications was not performed and directly followed by the addition of RLT buffer and ethanol. All samples were eluted with 16 μL pre-warmed RNase-free water to increase the final RNA concentration. Total RNA concentration and quality were determined using a high-sensitivity RNA screen type assay (Agilent) on a 4200 TapeStation System (Agilent).

#### T cell receptor library preparation and sequencing

Immediately after total RNA isolation, cDNA was generated using the Ion Torrent NGS Reverse Transcription Kit (Thermo Fisher Scientific). The maximum RNA input was loaded for cDNA synthesis to capture a large pool of T cell clones. Next-Generation Sequencing (NGS) libraries were prepared using the Oncomine TCR Beta-SR RNA Assay (Thermo Fisher Scientific), according to the manufacturer’s instructions. Amplified and barcode-ligated libraries were purified using AMPure XP Reagent (Beckman Coulter). The library quality check was performed using the High Sensitivity D1000 Screen Tape Assay (Agilent) on a 4200 TapeStation System (Agilent) and quantified using the Ion Universal Library Quantitation Kit (Thermo Fisher Scientific). The library pool was prepared by combining equal volumes of libraries at a concentration of 50 pmol/L and loaded into Ion 550 Chip (Thermo Fisher Scientific). The libraries were sequenced using an Ion GeneStudio S5 Prime Sequencer (Thermo Fisher Scientific). FASTQ files were generated using IonTorrent Server (Thermo Fisher Scientific).

#### TCR alignment and annotation of bulk TCR-seq

Read extraction and clonality counts were obtained from the FASTQ files using MiXCR v4.4.1 with the implemented presets for Oncomine TCR Beta-SR RNA Assay.^61^ Count tables were organized and explored using Immunarch R package.^56^ We determined the log2 counts per million (CPM) and log2-fold changes in the shared TCR sequences between the bulk TIL product and the positively sorted population. Only clones that were shared between both groups and positively enriched (logFC >0) in the sorted populations were classified as tumor-reactive. Tumor reactivity was verified in pre- or post-ACT samples using NeoTCR signatures or co-expression of tumor-reactivity signatures, exhaustion markers, and *CXCL13*, as described in the literature, as specific for tumor-reactive T cells.^21^^,^^22^^,^^23^^,^^62^

### Quantification and statistical analysis

#### Statistical methods

The R package geom_signif was used to evaluate statistical significance.^63^ Throughout the study, n denotes the number of individual patients, which represent the biological replicates; the exact n for each analysis is given in the corresponding figure legend and in the Results. For single-cell analyses, the number of cells or clonotypes per sample is reported in Table S2 and S3. For each quantified panel, the measure of center and the measure of dispersion are defined in the figure legend together with the statistical test and significance threshold. Cells and samples were excluded only according to the quality-control criteria described above or if too few cells were available for reliable conclusions.

### Additional resources

This study is associated with the clinical trial registered at ClinicalTrials.gov under identifier NCT04165967 (https://clinicaltrials.gov/study/NCT04165967).

## Supplemental information

Document S1. Figures S1–S7 and Tables S1–S3

Table S4. Cluster marker genes, differential gene expression results, and subtype-specific signature gene lists for tumor-reactive CD8^+^ and tumor-responsive CD4^+^ T cells, related to Figures 1, 2, 4, 6, and 7 (A) CD8_Cluster: marker genes defining each CD8^+^ T cell cluster. (B) CD8_DGE_tumor_reactive: genes differentially expressed between tumor-reactive and bystander CD8^+^ T cells. (C) CD8_DGE_reactive_IntermediatePr: genes differentially expressed between tumor-reactive and bystander CD8^+^ T cells in intermediate products. (D) CD8_reactive HLA-II sig: genes defining the tumor-reactive HLA-II signature. (E) CD8_reactive KLF2 sig: genes defining the tumor-reactive KLF2 signature. (F) CD4_Cluster: marker genes defining each CD4^+^ T cell cluster. (G) CD4_DGE_tumor_reactive: genes differentially expressed between tumor-reactive and bystander CD4^+^ T cells, with the subset used to build the tumor-responsive signature indicated. (H) CD4_DGE_responsive_Intermediate: genes differentially expressed between tumor-responsive and bystander CD4^+^ T cells in intermediate products. (I) CD4_responsive Tex sig: genes defining the tumor-responsive Tex signature. (J) CD4_responsive Tfh sig: genes defining the tumor-responsive Tfh signature. (K) Type17_signature: genes defining the type 17 T cell signature.

Table S5. Prior systemic anticancer therapy for each patient before TIL therapy, related to STAR Methods (A) PreTreatment: number and type of prior lines of systemic anticancer therapy per patient (UPN), including anti-PD-1 monotherapy; anti-CTLA-4 monotherapy; combined anti-PD-1/CTLA-4, BRAF, and MEK inhibitors; experimental therapy; and chemotherapy.

Document S2. Article plus supplemental information
