# Supplementary material for: Single-cell tracking reveals tumor-reactive T cell plasticity during melanoma TIL therapy
Source: Cell Rep Med. 2026 Sep 15;7(10):103051. doi: 10.1016/j.xcrm.2026.103051 (PMC13710383; doi:10.1016/j.xcrm.2026.103051)
Supplement: Supplementary file 1 [file mmc1.pdf]

Cell Reports Medicine, Volume *7*

Supplemental information

Single-cell tracking reveals tumor-reactive

T cell plasticity during melanoma TIL therapy

Michael T. Sandholzer, Clara Serger, Alessia G. Liner, Sarp Uzun, David König, Helen Thut, Reto Ritschard, Jonas D. Fürst, Andreas Zingg, Natalia Rodrigues Mantuano, Benjamin Kasenda, Katharina Glatz, Elisabeth A. Kappos, Matthias Matter, Andreas Holbro, Frank Stenner, Jakob Passweg, Nina Khanna, Lukas Jeker, Mascha Binder, Alfred Zippelius, and Heinz Läubli

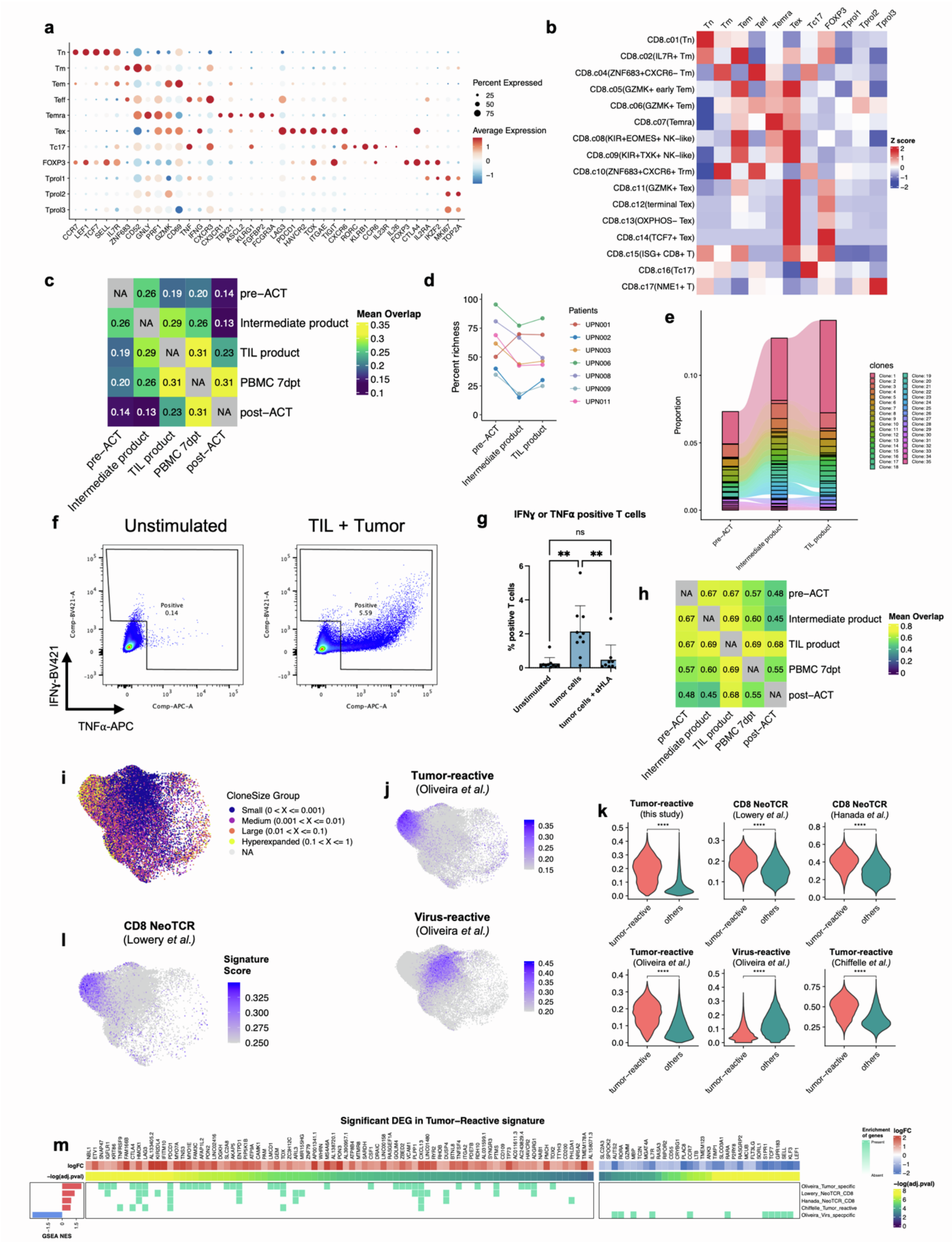

Figure S1. Cluster annotation, clonal overlap and functional validation of the tumor-reactivity assay in CD8$^+$ T cells, related to Figure 1. a) Dot plot showing the expression of selected marker genes used for cluster annotation. **b)** Heatmap depicting the signature mapping used for cluster annotation. Signatures were derived from Zheng *et al.*[1] **c)** Heatmap depicting mean TCR overlap of CD8$^+$ T cells between samples. **d)** Clonal diversity as percent richness between pre- and post-ACT. **e)** Alluvial plot highlighting clonal hierarchy as proportions of top clones during TIL expansion.

f) Example gating used for sorting of TNFα or IFNɣ positive populations. **g)** Bar plot indicating TNFα or IFNɣ positive fractions per condition. Mean ± SD. Statistical significance was assessed using Wilcoxon signed-rank test. **h)** Heatmap showing mean clonal overlap of tumor-reactive subpopulation across timepoints. **i)** Clonal expansion highlighted on UMAP. **j)** Signature score of tumor-reactive (top) and virus-reactive (bottom) T cells by Oliveira *et al.*[2] projected onto UMAP of tumor derived T cells. **k)** Signature score of selected signatures comparing identified tumor-reactive clones with remaining T cells in tumor derived samples. Statistical significance was assessed using Wilcoxon rank-sum test and Bonferroni Correction. **l)** Signature score of neoantigen-reactive $CD8^+$ T cells by Lowery *et al.*[3] projected onto UMAP of tumor derived T cells. **m)** Comparison of top differential expressed genes from $CD8^+$ tumor-reactive T cells with published signatures. * $p \leq 0.05$; ** $p \leq 0.01$; *** $p \leq 0.001$; **** $p \leq 0.0001$.

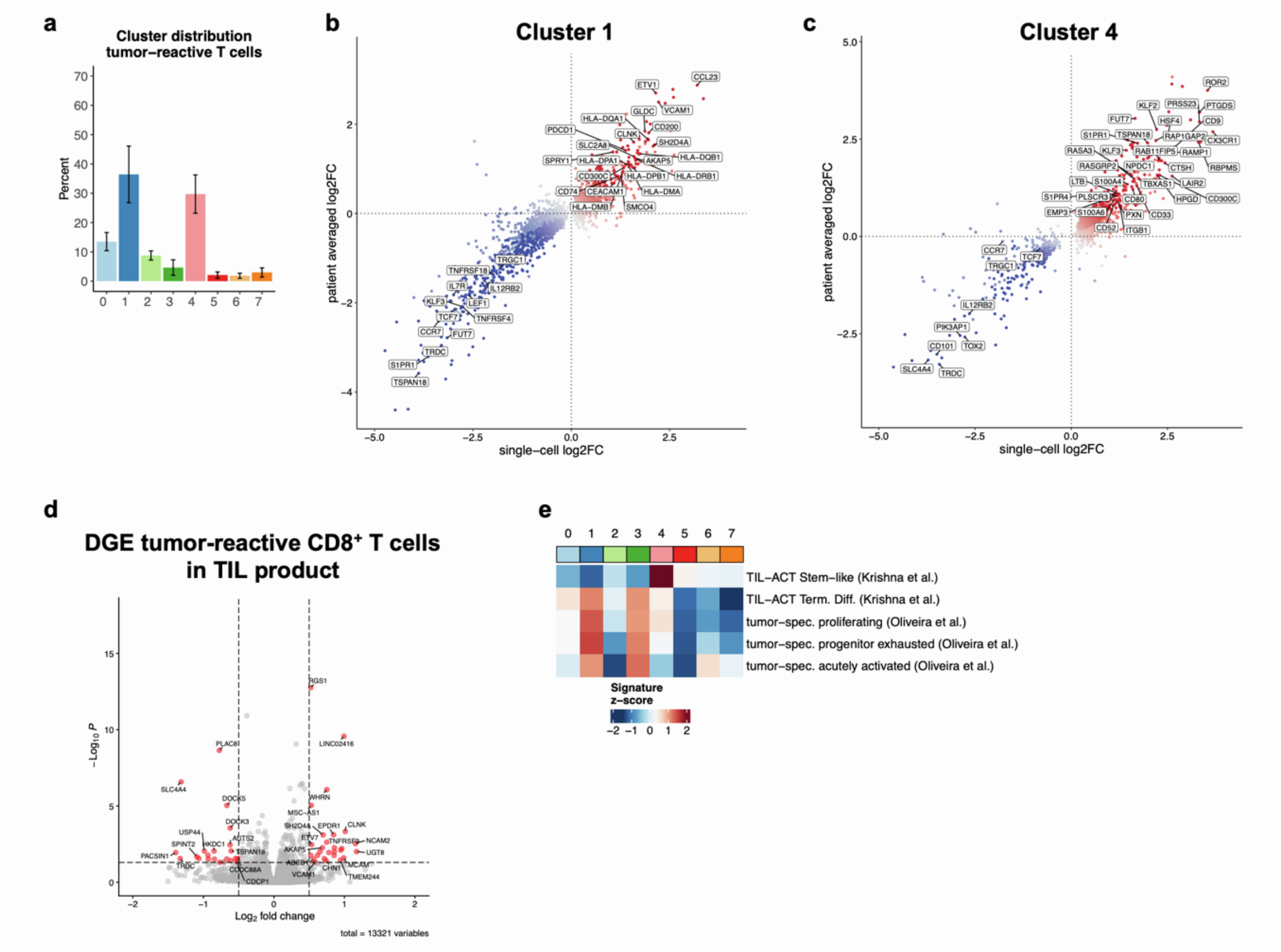

Figure S2. Differential gene expression and gene set enrichment underlying the HLA-II high and KLF2 high tumor-reactive $CD8^+$ states in intermediate and TIL products, related to Figure 2. a) Cluster distribution of tumor-reactive T cells in clusters of intermediate products. **b, c)** Differential gene expression comparing tumor-reactive $CD8^+$ subpopulations in corresponding cluster with remaining T cells in intermediate products using patient bulked comparison and single-cell comparison. Genes significantly upregulated in both comparisons were highlighted in dark color and genes significantly upregulated only in single-cell comparison in light color. **d)** Differential gene expression of tumor-reactive $CD8^+$ T cells in TIL products. **e)** Gene set enrichment of published signatures across identified clusters from intermediate products.

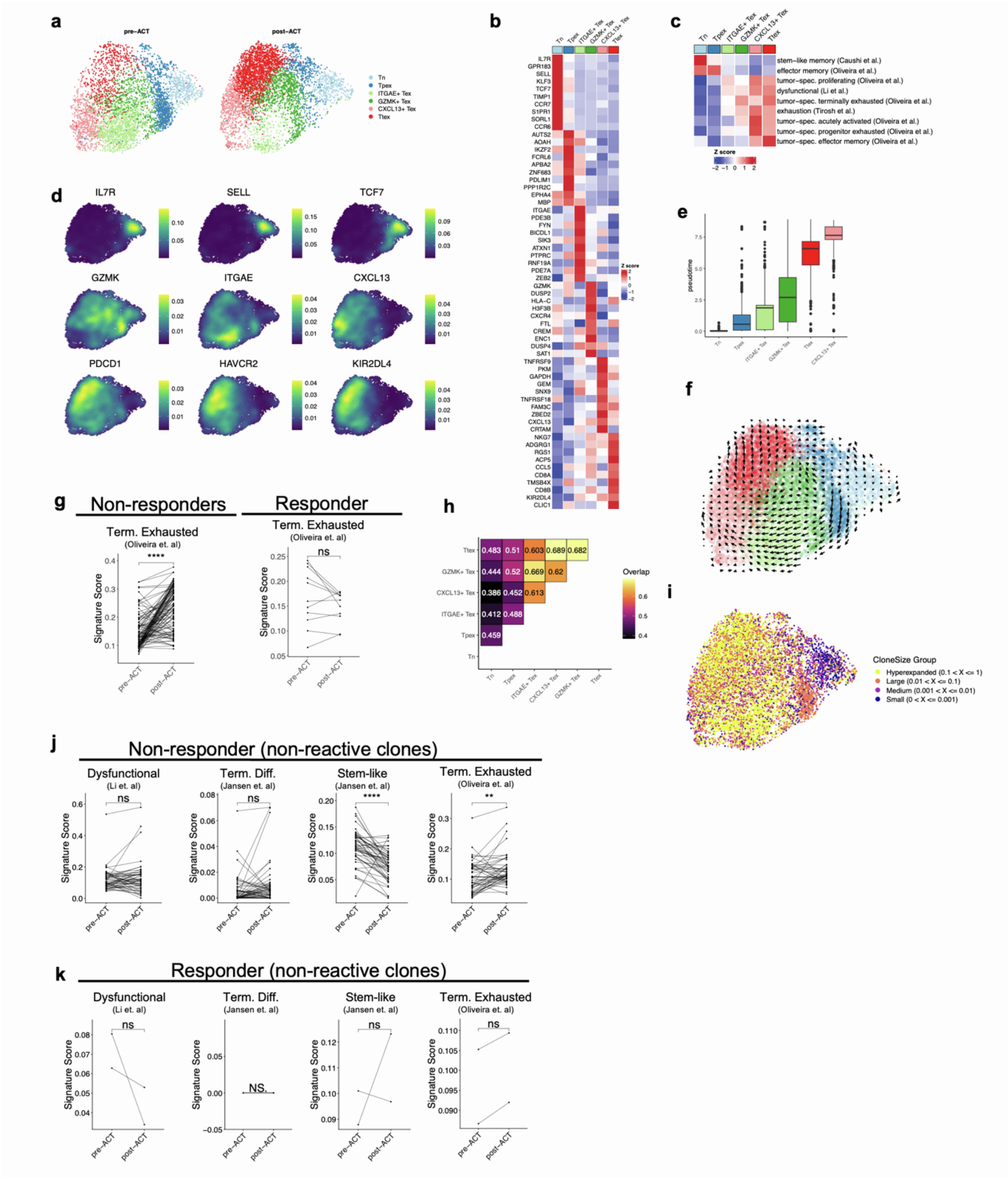

Figure S3. Marker gene expression, pseudotime, RNA velocity and clonal overlap supporting the tumor-reactive $CD8^+$ T cell states in pre- and post-ACT lesions, related to Figure 3. a) UMAP visualization of the cell states split into pre- and post-ACT lesions. **b)** Heatmap showing the top genes differentially expressed between clusters. **c)** Gene set enrichment of published signatures across identified clusters of tumor-reactive $CD8^+$ T cells. **d)** UMAP plots showing the expression levels of selected marker genes. **e)** Pseudotime values per cell grouped by clusters. **f)** RNA velocities calculated based on unspliced vs. spliced RNA embedded in UMAP. **g)** Signature scores of published signatures comparing mapped clones detected in pre- and post-ACT lesions of Rs and NRs[2]. Statistical significance was assessed using Wilcoxon rank sum test. **h)** Heatmap depicting clonal overlap between clusters. **i)** Expansion state highlighted on UMAP representation. **j & k)** Signature scores of published signatures comparing non-reactive clones of pre- and post-ACT lesions of Rs and NRs[4,5]. Statistical significance was assessed using Wilcoxon signed-rank test. * $p \leq 0.05$; ** $p \leq 0.01$; *** $p \leq 0.001$; **** $p \leq 0.0001$.

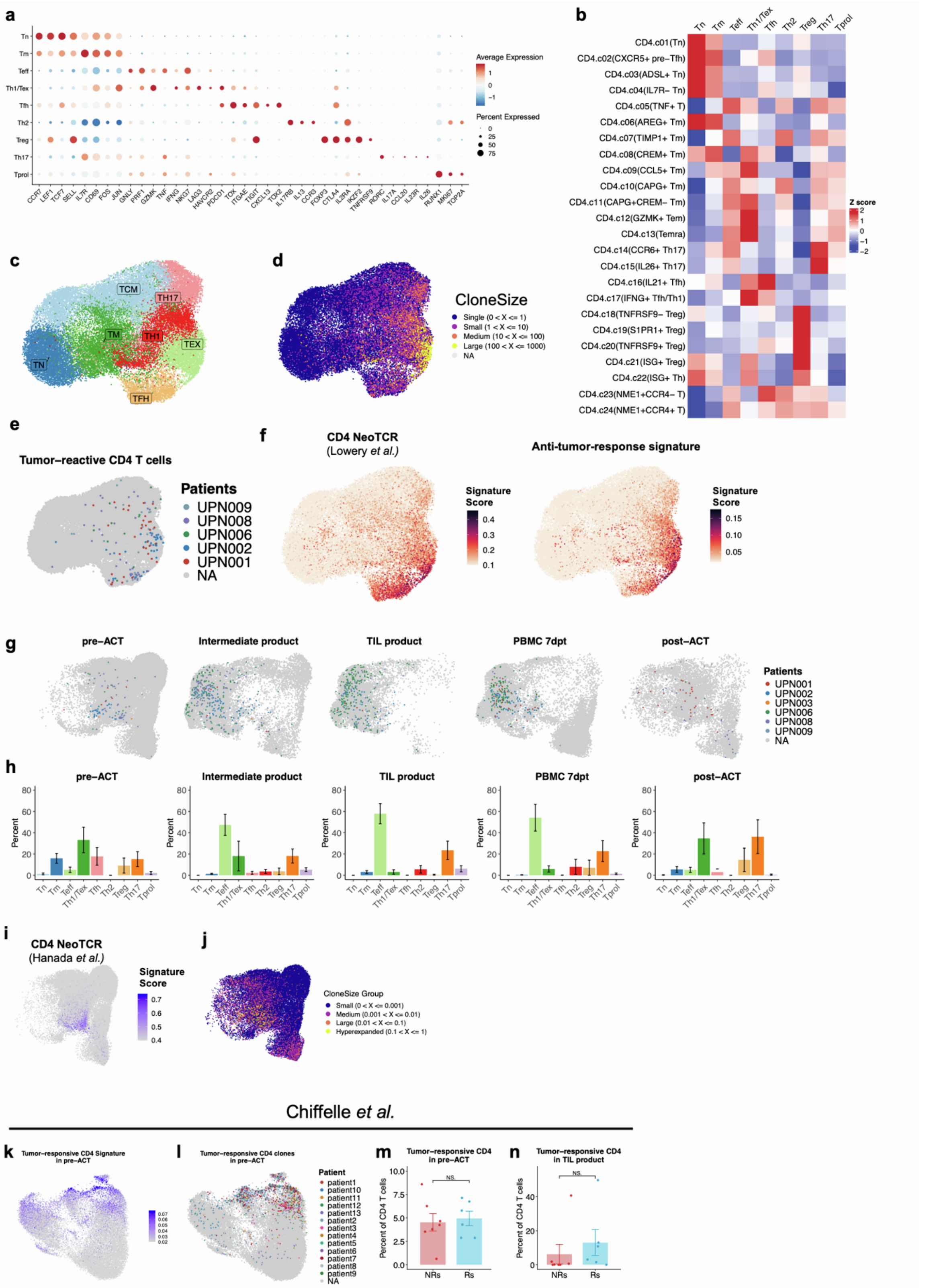

Figure S4. Cluster annotation, derivation and external validation of the tumor-responsive $CD4^+$ T cell signature, related to Figure 4. a) Dot plot showing the expression of selected marker genes used for cluster annotation. **b)** Heatmap depicting the signature mapping used for cluster annotation. Signatures were derived from Zheng *et al.*[1]. **c)** Subclustering of conventional $CD4^+$ T cells of pre-ACT and post-ACT samples. TN, naïve T cells; TCM, central memory T cells; TM, memory T cells; TH1, T helper 1 cells; TH17 T helper 17 cells; TFH, follicular helper T cells; TEX, exhausted T cells. **d)** Clonal expansion highlighted on subclustering of conventional $CD4^+$ T cells. **e)** Tumor-reactive $CD4^+$ T cells highlighted on subclustering of

conventional CD4+ T cells. **f)** CD4 Neoantigen-reactive T cell signature by Lowery *et al.*[3] and Anti-tumor-response signature created in this study highlighted on UMAP. **g)** Tumor-reactive CD4+ T cells highlighted in the UMAP representation at collected time points. **h)** Bar plots showing the mean CD4+ T cell composition of the tumor-reactive subpopulation per time point. Error bars indicate SEM. **i)** Signature score of the CD4+ neoantigen-reactive T cell signature by Hanada *et al.*[6]. **j)** Clonal expansion highlighted in UMAP of pre-ACT CD4+ T cells. **k)** Featureplot of our tumor-responsive CD4+ signature on pre-ACT CD4+ T cells of the Chiffelle dataset[7]. **l)** Identified tumor-responsive CD4+ clones on pre-ACT CD4+ T cells of the Chiffelle dataset[7]. **m)** Percent of tumor-responsive CD4+ clones in pre-ACT pre-ACT CD4+ T cells of the Chiffelle dataset[7]. **n)** Percent of tumor-responsive CD4+ clones in TIL product CD4+ T cells of the Chiffelle dataset[7].

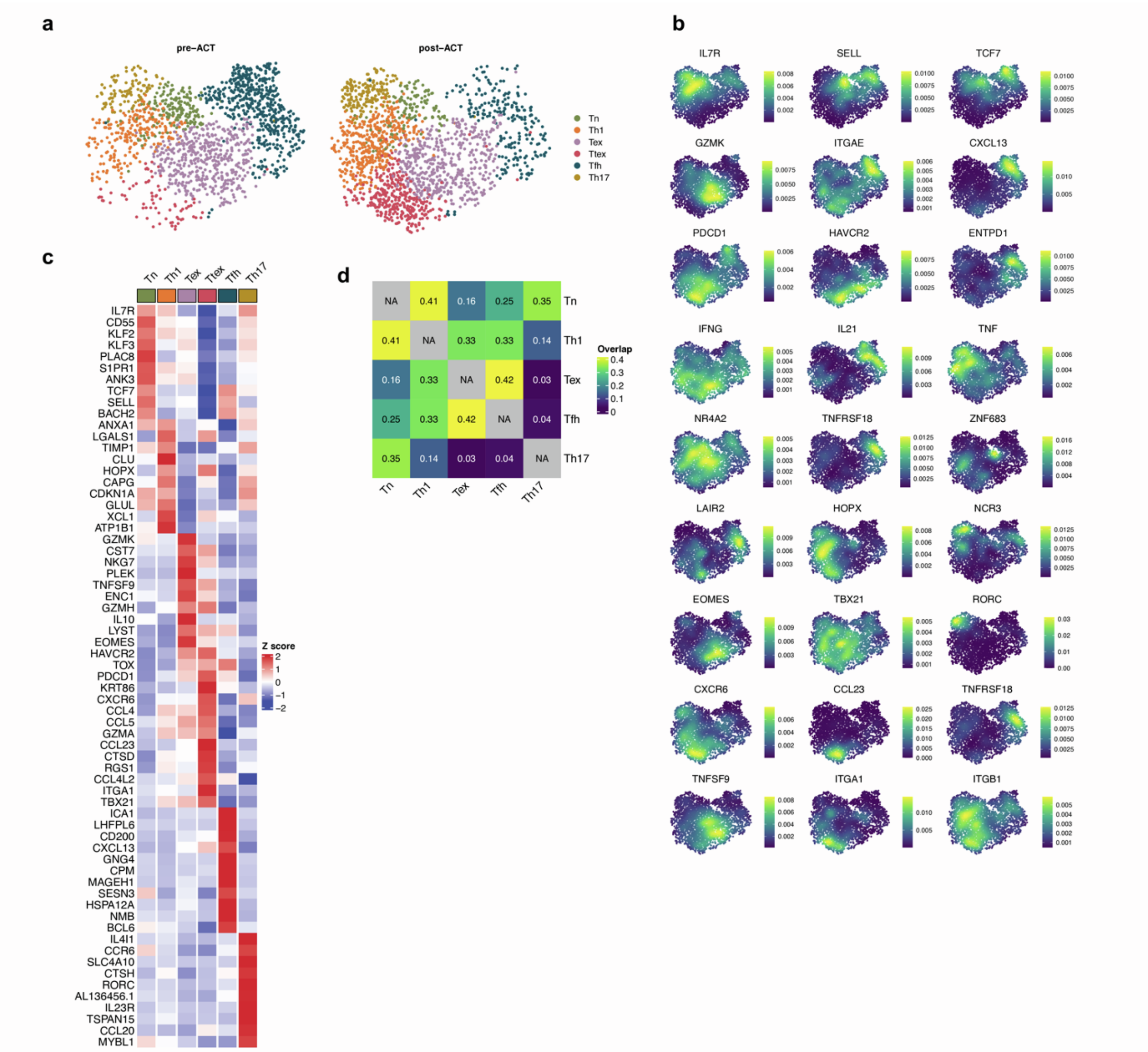

Figure S5. Marker gene expression, cluster-defining genes and clonal overlap for tumor-responsive CD4+ T cells in pre- and post-ACT lesions, related to Figure 5. a) UMAP visualization of tumor-responsive CD4+ T cell states split into pre- and post-ACT lesions. **b)** UMAP plots showing the expression levels of selected marker genes. **c)** Heatmap showing the top genes differentially expressed between clusters **d)** Heatmap presenting clonal overlap between clusters.

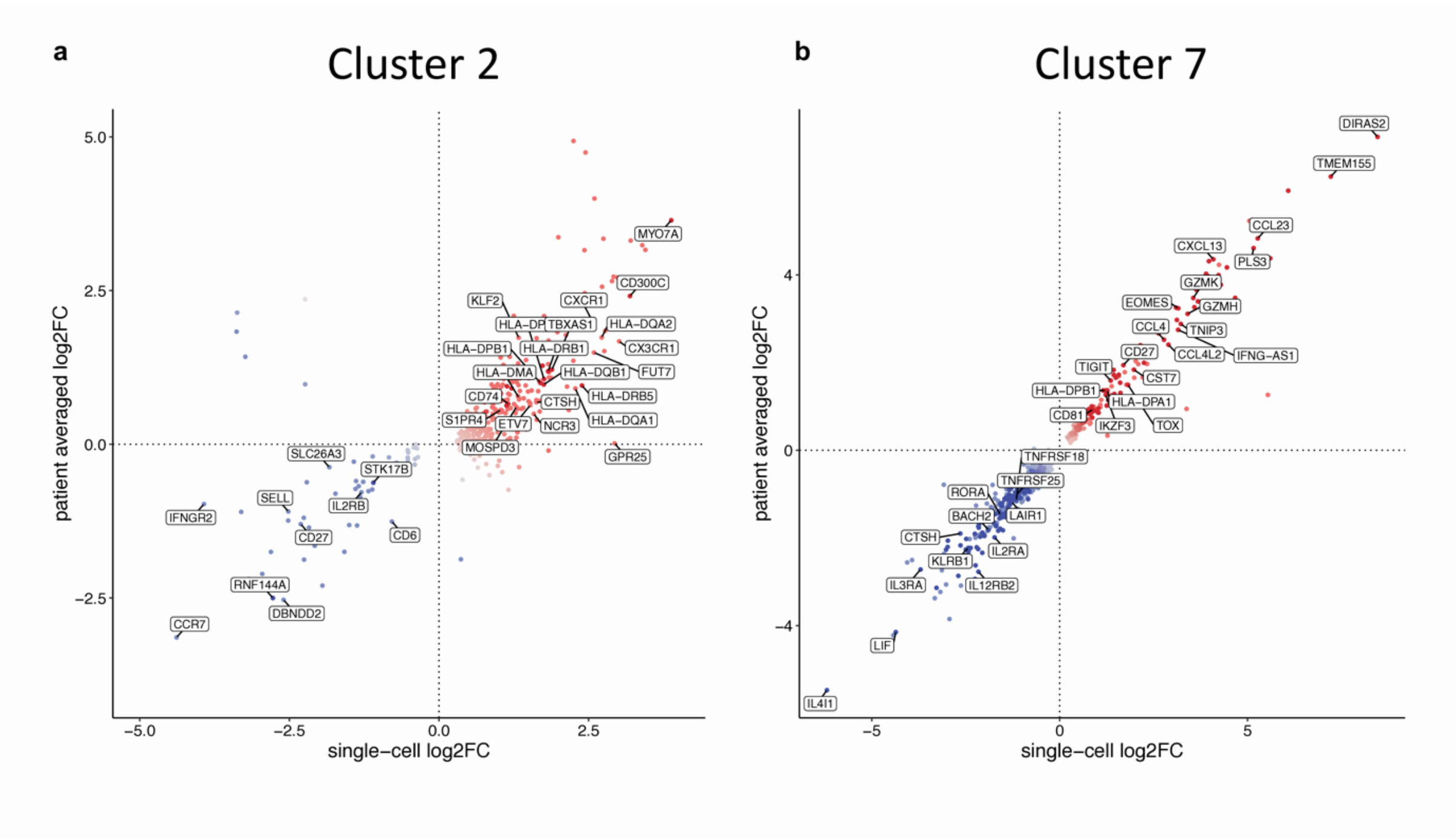

Figure S6. Differential gene expression defining the reinvigorated Tfh and non-reinvigorated Tex clusters in intermediate products, related to Figure 6. a-b) Differential gene expression comparing tumor-responsive $CD4^+$ subpopulations in corresponding cluster with remaining T cells in intermediate products using patient bulked comparison and single-cell comparison. Genes significantly upregulated in both comparisons were highlighted in dark color and genes significantly upregulated only in single-cell comparison in light color.

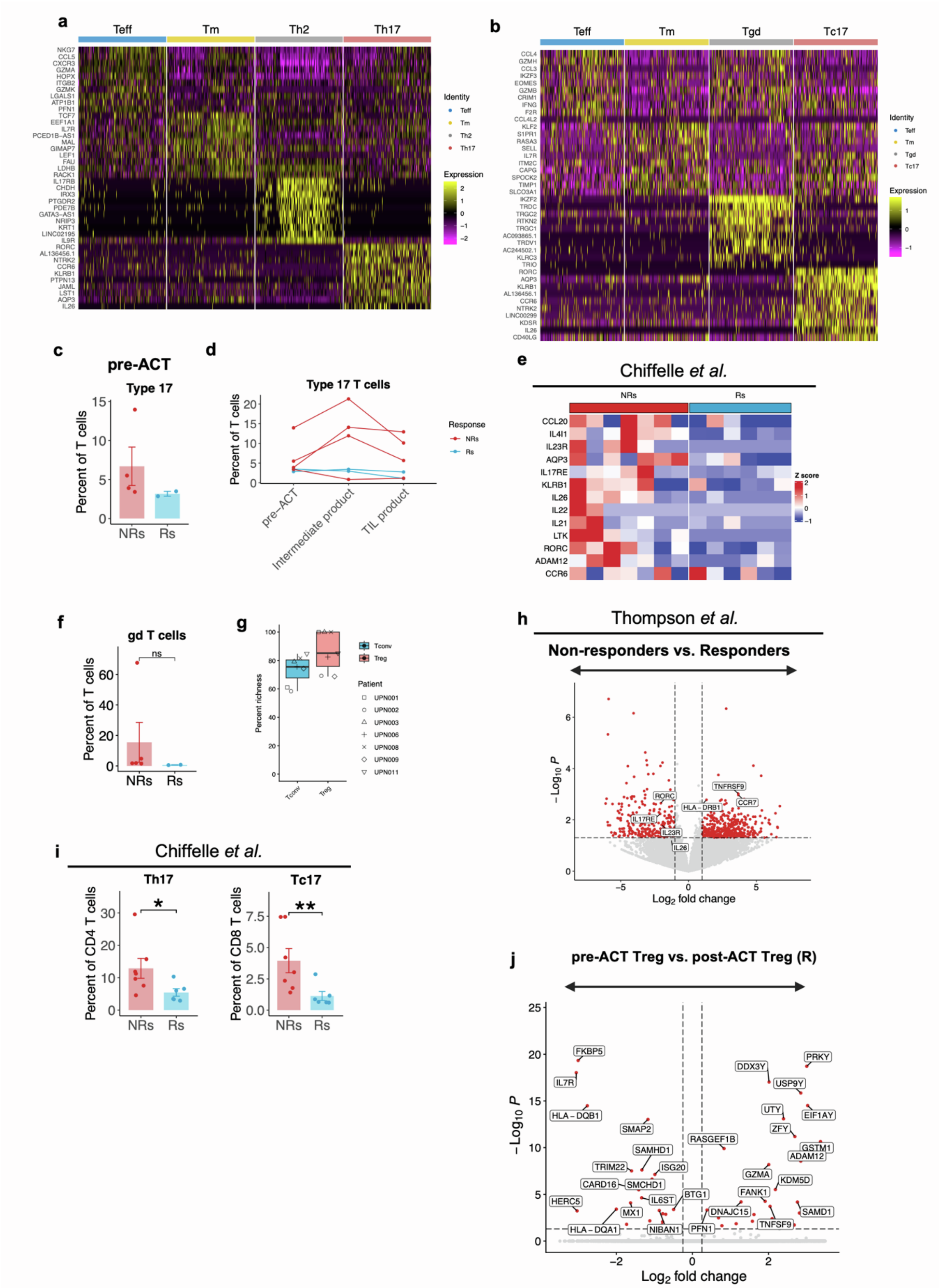

Figure S7. Cluster-defining genes, Type 17 T cell frequencies across validation cohorts and Treg analyses supporting the association with TIL therapy resistance, related to Figure 7. a) Heatmap showing the top genes differentially expressed between clusters of CD4 TIL products. **b)** Heatmap showing top genes differentially expressed between clusters of CD8 TIL product. **c)** Mean frequency of Type 17 T cells in pre-ACT lesions in the dataset by Chiffelle *et al.*[7]. Error bars indicate SEM. Statistical significance was assessed using Wilcoxon rank sum test. **d)** Frequency change of Type 17 cells during TIL expansion. **e)** Heatmap showing the expression of selected Th17 and Tc17 marker genes expressed in the dataset by

Chiffelle *et al.*[7]. **f)** Percent of gdT cells in TIL products of NRs and Rs. Statistical significance was assessed using Wilcoxon rank sum test. **g)** Clonal diversity as Percent richness in conventional T cells (Tconv) and Tregs per patient. **h)** DGE comparing CD4 TIL products of NRs and Rs on the dataset by Thompson *et al.*[8]. **i)** Mean frequency of Th17 cells in CD4 TIL and Tc17 cells in CD8 TIL products in the dataset by Chiffelle *et al.*[7]. Error bars indicate SEM. Statistical significance was assessed using Wilcoxon rank sum test. * $p \leq 0.05$; ** $p \leq 0.01$ **j)** Differential gene expression comparing Tregs from pre-ACT and post-ACT in Rs.

Table S1. Clinical and sample metadata for the seven patients included in the single-cell experiment, related to Figure 1.

Patient	Response	Gender	Transfused cells	time to post-ACT.1	time to post-ACT.2
UPN001	PR	male	74.25 x 10e9	21 weeks	62 weeks
UPN002	PD	male	50 x 10e9	NA	NA
UPN003	PR	female	70 x 10e9	NA	NA
UPN006	SD	male	57 x 10e9	6 weeks	15 weeks
UPN008	SD	female	66.25 x 10e9	11 weeks	33 weeks
UPN009	SD	female	68.25x 10e9	31 weeks	31 weeks
UPN011	PD	male	63.25 x 10e9	8 weeks	NA

Table S2. Number of high-quality T cells recovered per patient and timepoint after quality-control filtering, related to Figure 1.

Patient	pre-ACT	Intermediate product	TIL product	PBMC 7dpt	post-ACT.1	post-ACT.2	PBMC_Ctrl
UPN000	NA	NA	NA	NA	NA	NA	13176
UPN001	3184	1864	6396	10593	6114	2232	NA
UPN002	2806	7590	9034	11379	NA	NA	NA
UPN003	5796	8401	8008	17328	NA	NA	NA
UPN006	12466	8222	11289	14909	424	331	NA
UPN008	28878	12572	14562	16972	7128	5627	NA
UPN009	9824	6352	12977	10684	13644	8046	NA
UPN011	6149	10932	18634	2305	6900	NA	NA

Table S3. Number of T cells with a productive TCRβ CDR3 sequence per patient and timepoint after quality-control filtering, related to Figure 1.

Patient	pre-ACT	Intermediate product	TIL product	PBMC 7dpt	post-ACT.1	post-ACT.2	PBMC_Ctrl
UPN000	NA	NA	NA	NA	NA	NA	11981
UPN001	2790	1491	5725	9084	5208	1615	NA
UPN002	1648	2053	1504	4718	NA	NA	NA
UPN003	5086	6531	5926	14394	NA	NA	NA
UPN006	11832	7234	10189	13255	391	287	NA
UPN008	26113	10101	12094	13100	6531	4428	NA
UPN009	8302	4826	10482	9474	10128	6161	NA
UPN011	4613	7653	15422	2087	5936	NA	NA

References

1. Zheng, L., Qin, S., Si, W., Wang, A., Xing, B., Gao, R., Ren, X., Wang, L., Wu, X., Zhang, J., et al. (2021). Pan-cancer single-cell landscape of tumor-infiltrating T cells. Science *374*, abe6474. 10.1126/science.abe6474.
2. Oliveira, G., Stromhaug, K., Klaeger, S., Kula, T., Frederick, D.T., Le, P.M., Forman, J., Huang, T., Li, S., Zhang, W., et al. (2021). Phenotype, specificity and avidity of antitumour CD8(+) T cells in melanoma. Nature *596*, 119-125. 10.1038/s41586-021-03704-y.
3. Lowery, F.J., Krishna, S., Yossef, R., Parikh, N.B., Chatani, P.D., Zacharakis, N., Parkhurst, M.R., Levin, N., Sindiri, S., Sachs, A., et al. (2022). Molecular signatures of antitumor neoantigen-reactive T cells from metastatic human cancers. Science *375*, 877-884. 10.1126/science.abl5447.
4. Li, H., van der Leun, A.M., Yofe, I., Lubling, Y., Gelbard-Solodkin, D., van Akkooi, A.C.J., van den Braber, M., Rozeman, E.A., Haanen, J., Blank, C.U., et al. (2019). Dysfunctional CD8 T Cells Form a Proliferative, Dynamically Regulated Compartment within Human Melanoma. Cell *176*, 775-789 e718. 10.1016/j.cell.2018.11.043.
5. Jansen, C.S., Prokhnevska, N., Master, V.A., Sanda, M.G., Carlisle, J.W., Bilen, M.A., Cardenas, M., Wilkinson, S., Lake, R., Sowalsky, A.G., et al. (2019). An intra-tumoral niche maintains and differentiates stem-like CD8 T cells. Nature *576*, 465-470. 10.1038/s41586-019-1836-5.
6. Hanada, K.I., Zhao, C., Gil-Hoyos, R., Gartner, J.J., Chow-Parmer, C., Lowery, F.J., Krishna, S., Prickett, T.D., Kivitz, S., Parkhurst, M.R., et al. (2022). A phenotypic signature that identifies neoantigen-reactive T cells in fresh human lung cancers. Cancer Cell *40*, 479-493 e476. 10.1016/j.ccell.2022.03.012.
7. Chiffelle, J., Barras, D., Pétremand, R., Orcurto, A., Bobisse, S., Arnaud, M., Auger, A., Rodrigo, B.N., Ghisoni, E., and Sauvage, C. (2024). Tumor-reactive T cell clonotype dynamics underlying clinical response to TIL therapy in melanoma. Immunity.
8. Thompson, B., Strange, A., Amato, C.M., Hester-McCullough, J., Sarnaik, A.A., Weber, J.S., and Woods, D.M. (2023). CD4 Phenotypes Are Associated with Reduced Expansion of Tumor-Infiltrating Lymphocytes in Melanoma Patients Treated with Adoptive Cell Therapy. J Immunol *211*, 735-742. 10.4049/jimmunol.2300250.
